# Synergistic approaches for hexapod mobility: comparative evaluation of structure, navigation, and control strategies on challenging terrains

**DOI:** 10.1038/s41598-025-34857-9

**Published:** 2026-01-22

**Authors:** Sivayazi Kappagantula, Giriraj Mannayee

**Affiliations:** 1 Manipal Institute of Technology, Manipal Academy of Higher Education, Manipal, India; 2 Department of Design and Automation, School of Mechanical Engineering, Vellore Institute of Technology, Vellore, India

**Keywords:** Hexapod robot, Structural stress analysis, Autonomous navigation, Adaptive joint control, Multi terrain navigation, Engineering, Mathematics and computing

## Abstract

The study delivers a cohesive system that combines structural stress investigation, navigational planning evaluation, and adaptive joint control to optimize hexapod effectiveness on hills, stairs, and uneven surfaces. The robot was developed through the iterative drafting technique and designed by assigning in PLA material. Structural examination with Finite Element Analysis (FEA) under 10 N and 20 N forces demonstrated a positive stress allocation and a safety factor of 2.8, combining compact development with durability. In the ROS/Gazebo exploration investigations utilizing global planners like A*, Dijkstra, RRT, and Artificial Potential Field (APF) in combination with a PID-driven local planner, A* as well as Dijkstra developed nearly the best pathways with 100% accuracy. This cut down on route variation by about 17% in comparison to RRT. RRT established confident that the exploration was always the same, but it established paths that were more lengthy and less smooth. APF, on the contrary, made paths that were smooth but less reliable due to the local minima. Adaptive synchronization for joint control quantitatively provided an improvement in joint angle stability, reducing oscillatory deviations by 12% and displacement errors by 15% relative to baseline controllers. The core novelty within this approach is the integrative methodology that will inherently synergize finite element structural analysis, comparative path planning, and Adaptive joint synchronization: presenting a comprehensive optimization strategy, new to hexapod robotics. Together, these advances allow for robust and efficient real-world deployment of hexapods. Future work will extend to hybrid learning-based planning, and sensor-driven dynamic adaptation.

## Introduction

**Fig. 1:**
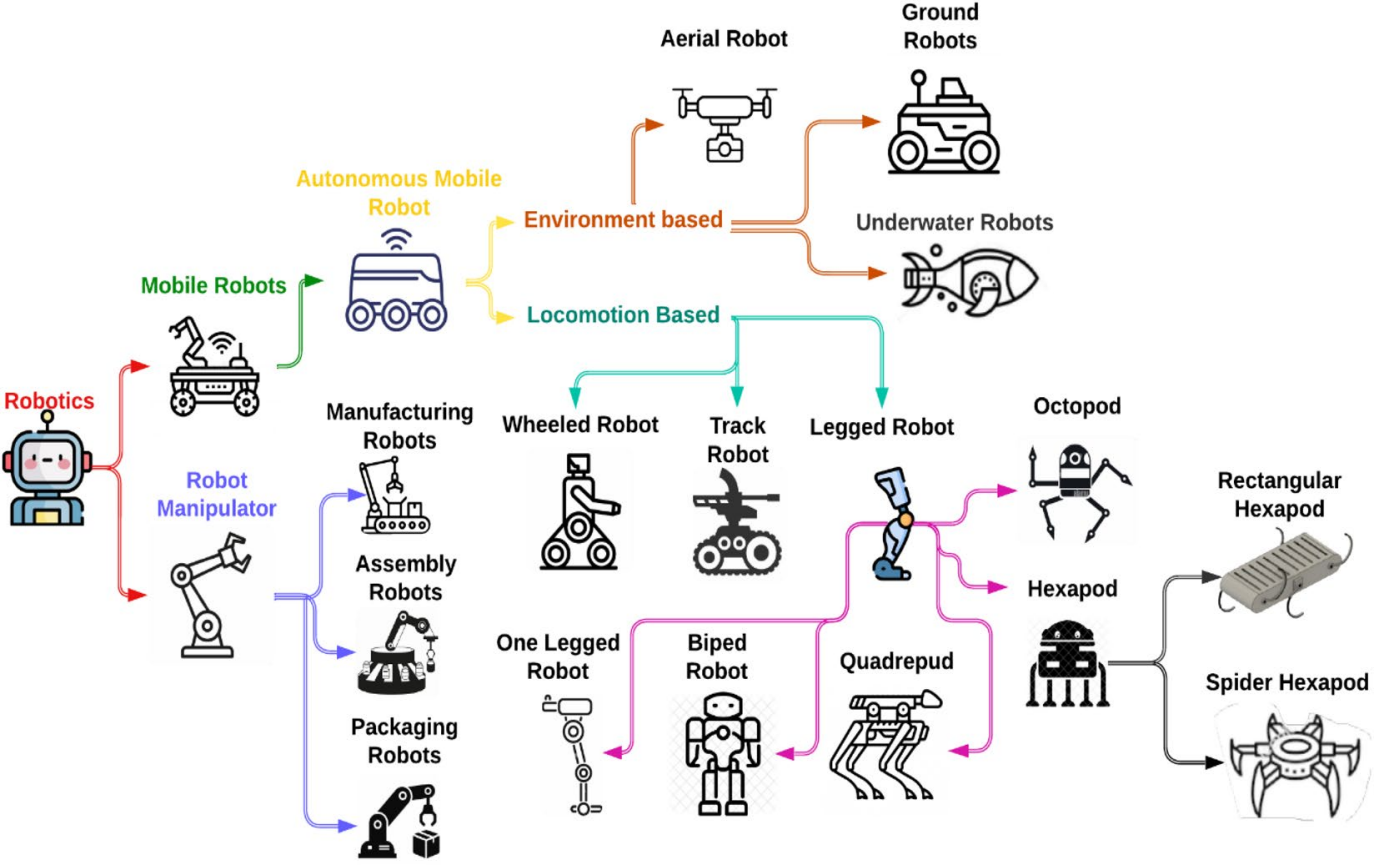
Classification of robots^10^.

Industrial operations like production and assembly are central to robots. Wheeled robots provide velocity on even surfaces, whereas legged robots provide capability on rough ground and are thus appropriate for rescue and military applications^1–5^. Legged robots, unlike detouring wheeled systems, step or hop over obstacles^6^. Hexapods (Fig. 1), drawn from bio-inspired six-legged structures, are stable, durable, and versatile^7,8^. Although more energy-hungry and more difficult to stabilize, they allow sideways and inclined locomotion rare in other robots^9^.

Locomotion relies on gait coordination and path planning through terrains (flat, slopes, stairs) with traction, elevation adjustment, and stress reduction challenges^11,12^ Structural stability, effective path planning (A*, Dijkstra’s, RRT, APF), and adaptive joint control for real-time stability are the main factors. Materials & Stress: Little stress distribution analysis; lightweight polymers hardly researched^13,14^. A* and Dijkstra’s precise information but expensive; RRT is fast but not reliable; constrained multi-terrain comparisons^15–19^. Limited quantitative joint-angle control research on stability information^19,20^. The present work employs FEA for stresses, analyzes planners (APF, RRT, Dijkstra’s, A*), and explores adaptive joint control for increasing hexapod efficiency and longevity. Advances are telescopic legs^13,14^ stable attitude control^14^, inverted pendulum models^15^, robots at the centimeter scale^16^, multimodal designs^17^, and predictive foot-force algorithms^18^. Material studies demonstrate carbon fibre and aluminium are more stress resistant than PLA^19,21,22^. Trade-offs in path planning emphasize the requirement for hybrid approaches^21,23–25^. Joint coordination increases the stability as well as energy economic performance^26^.

The latest developments in hexapod autonomous systems have demonstrated significant advances in mechanical design, path planning^2–4^, and control techniques. Finite element approaches (FEM) for structural evaluation make certain that structures are strong enough to handle real-world loads. There are many different path planning algorithms that have been investigated for legged robot mobility, which include A*, Dijkstra, Rapidly exploring Random Tree (RRT), and Artificial Potential Field (APF). Each one has advantages as well as disadvantages^7–9^. Adaptive joint controls inspired by biological locomotion boost balance and energy conservation, as discussed in^27^. Nonetheless, the current research predominantly examines each of these elements in individually. This paper addresses the gap by integrating these areas collectively into a single system of operation, which makes it easier to work on uneven surfaces. Key recent works relevant to this study include^28^, which explores adaptive control in legged robots^29^, which presents FEM analyses of legged robot structures and^30^, focused on path planning optimization under dynamic constraints.

Modern advances in hexapod technology have enhanced the design of machines, navigation methods, and control approaches; however, considerable constraints persist. Most of the research on material design focus on lightweight materials like PLA, for example, but they don’t do enough to look at dynamic loading and impact resistance. This represents an enormous void in the quest for robust but adaptable hexapod frame systems. There are quite numerous approaches of establishing a route, but each one has benefits as well as drawbacks. In particular, A* and Dijkstra are traditional approaches that find the most effective routes, but they cost considerably to employ. RRT determines paths, but they aren’t smooth as it ought to be, and Artificial Potential Field methods have challenges with local minima. This suggests that we must utilize computations that are a combination of different types or that learn from previous experiences to function effectively in complex situations. Adaptive controlling techniques based on physical movement patterns demonstrate great potential for improving joint stability and locomotion performance; however, numerous approaches have weaknesses in quantitative evaluation and comprehensive testing within structure and navigational frameworks. The present research proposes an integrated scheme that integrates computational analysis of structures, comparative path-planning evaluation, and real-time adaptive joint synchronization to address these interrelated issues. This framework presents a thorough optimization technique which increases the capabilities of hexapod robots in diverse terrain conditions.

The circumstances are growing more stable, nevertheless there remain shortcomings with material effectiveness, planning of paths, and control adaptation. To make locomotion over slopes, stairs, and rough terrain stable, efficient, and autonomous, integrated solutions must be used. Our framework combines structural, navigation, and control modules in a way that makes the system more adaptable to different terrains and more stable overall. This is different from previous comparative studies that looked at these areas separately. Adaptive synchronization here means controlling the gait and joints in a way that is in sync with each other, not a new algorithmic formula.

## Methodology

**Fig. 2:**
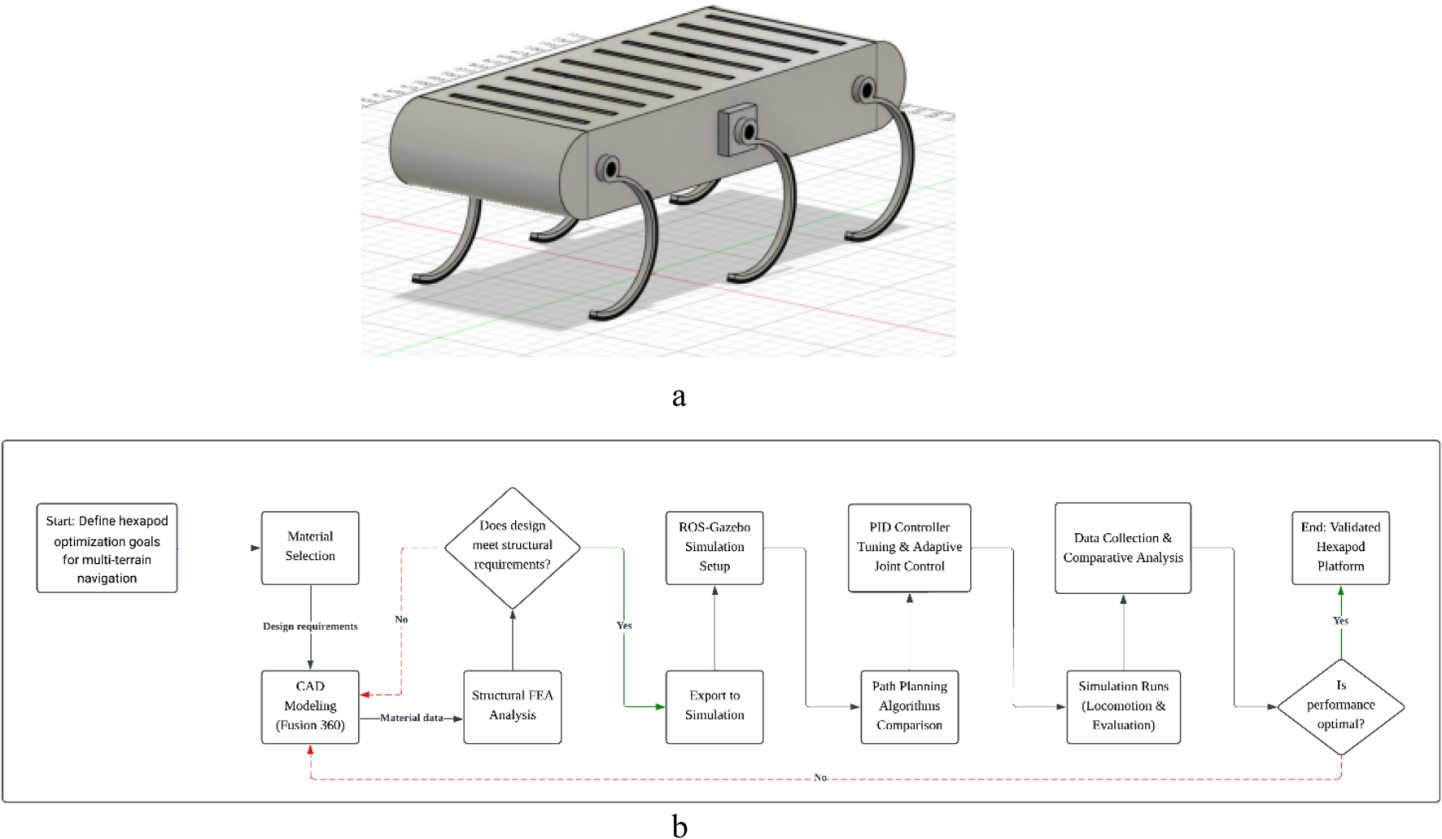
(**a**) CAD modelling for proposed hexapod. (**b**) Methodology flowchart illustrating the integrated workflow for hexapod optimization, spanning material selection, CAD modeling, structural FEA analysis, simulation setup in ROS-Gazebo, path-planning algorithm comparison, PID controller tuning with adaptive joint control, simulation-based performance evaluation, and iterative optimization decisions.

Figure 2a and b depicts the end-to-end process used in the study to optimize the hexapod robot for multi-terrain navigation. Starting from design goals, material selection, and CAD modeling in Fusion 360, the workflow includes structural verification via finite element analysis (FEA) with iterative feedback to design if requirements are unmet. The validated model is exported for dynamic simulation in ROS-Gazebo, where multiple path-planning algorithms are assessed quantitatively. PID controller tuning combined with adaptive joint control enhances locomotion stability. Simulation runs generate performance data which undergo comprehensive comparative analysis. The final optimization decision loop allows refinement to achieve the best balance of structural integrity and navigation performance, terminating when the optimal configuration is reached.

### Hexapod design and stress analysis

The hexapod was designed employing an iterative design process comprised of material selection, motor integration, URDF conversion, and simulation within ROS. The prototype featured in Fig. 2 integrates revisions to ensure the highest level of structural functionality.

#### Hexapod structure and material selection

The body (518 × 248 × 100 mm) was 3D-printed in lightweight PLA due to ease of manufacturability. Minimum thicknesses were 1 mm (top/sides) and 2 mm (bottom) to balance strength and weight.

The microcontroller, motors, and control mechanisms are housed within, with air slits provided for cooling. Support structures and reinforced joints were added to reduce deformation under load.

#### Finite element analysis (FEA)

Structural integrity was confirmed under 10N and 20N load tests. Stress concentration was noticed near joints, resulting in panel thickness adjustments and additional reinforcement. Stress levels were compared to state-of-the-art hexapods constructed using carbon fibre, aluminium, and high-strength steel, based on performance criteria including displacement, safety factor, and stress zones^19,21,22^.

#### Dynamic structural validation

To evaluate evolving durability over the fundamental static test situations involving 10 N and 20 N, a further quasi-static and sensitivity-controlled validation was conducted to simulate load variations during locomotion. The torso weight was altered in several ways according to the intended operational range and utilized in a couple of posture variations by moving the efficient support areas at the foot connecting points. This made it possible to mimic distinct movement periods and equilibrium stances. In all these scenarios, the greatest amount of stress coefficients and nodal regions motion stayed somewhere between 5 to 10% of the original equilibrium parameters. The safety coefficient for the PLA structure was always over 2.5, which means that it had adequate durability for anticipated conditions of service. We ran the modeling procedures many times employing the optimized mesh employed (global size of elements 1.5 mm, local 0.8 mm at joints) and determined that the ultimate stress variations consisted of roughly 0.3 MPa. This shows that the dimensional estimations were stable and reliable. Although an exhaustive intermittent dynamic modeling encompassing impact loads, shaking mechanisms, and time-dependent gait-induced forces exceeds the present scope, these geometric and reproducible analyses establish an optimistic framework for the stresses encountered during mobility, stair ascent, and slope navigation, while explicitly signalling the intended progression towards extensive changes finite element computation in subsequent investigations.

#### Safety factor calculation

The above setup ensured that stress distribution and safety factors were thoroughly evaluated for various payload scenarios.

The finite element analysis presented in this study focuses on static loading conditions (10 N and 20 N) to assess the structural integrity of the hexapod robot under constant loads. While this provides a baseline for material and design validation, dynamic loading scenarios including vibrations, impacts, and time-varying forces—will be addressed in future work to further enhance the robot’s robustness for real-world applications.

#### Path planning and navigation

Navigation in the ROS/Gazebo system was done via a combination of global path planning strategies and a PID-based local planner. Of the global planners, A* was used to generate near-optimal paths, albeit computationally expensive. Dijkstra’s algorithm provides exact solutions at the expense of excessive resource use. Rapidly-Exploring Random Trees (RRT), however, offer effective exploration of the world but tend to have paths with compromised smoothness. In the meantime, the Artificial Potential Field (APF) approach allows real-time collision avoidance, albeit vulnerable to obstacles like local minima^21,23–25^.

#### PID local planner

**Fig. 3:**
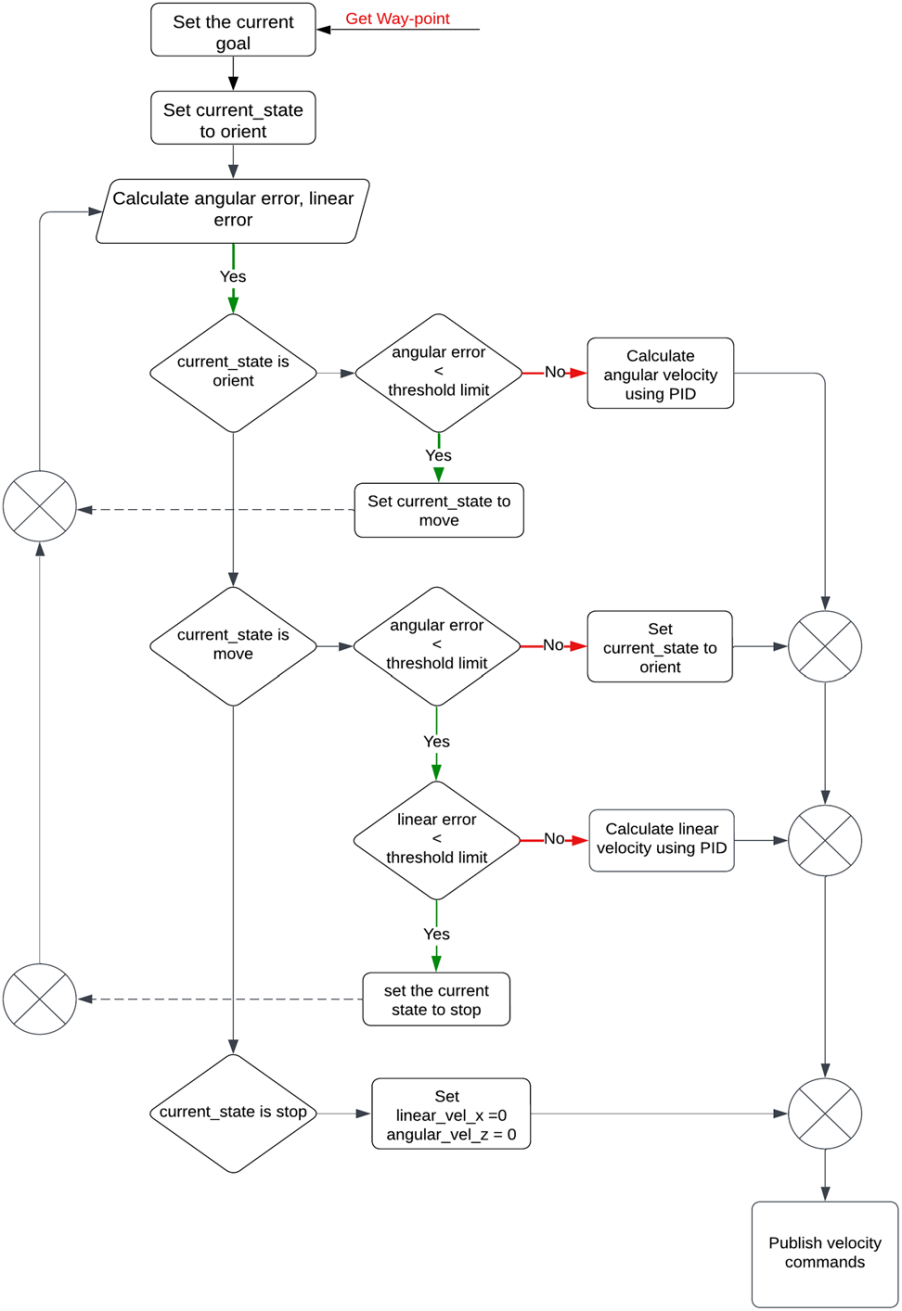
Description of process for PID control.

A PID controller in Fig. 3 guaranteed waypoint execution by state transitions (orient–move–stop). The controller subscribed to odometry, executed random waypoints sequentially after processing, and ensured static obstacles were avoided while ignoring dynamic obstacles. Pseudocode implementation proceeded with error-based corrections in orientation and distance.

Path planning algorithms were implemented within ROS-Gazebo. PID controllers facilitated waypoint tracking for the hexapod’s navigation, with tuning parameters derived through iterative testing aimed at minimizing trajectory deviation and oscillations.

PID values:

- Proportional gain $${K}_{p}=1.2$$
- Integral gain $${K}_{i}=0.01$$
- Derivative gain $${K}_{d}=0.1$$

These parameters achieved stable control, balancing responsiveness and overshoot reduction.

#### Evaluation metrics

- Time to reach goal
- Path smoothness (trajectory deviation)
- Success rate over terrains

#### Experimental protocol and statistical analysis

To guarantee highly accurate assessment of movement and measurement effectiveness, every single global strategy (A*, Dijkstra, RRT, and APF) was carried out in several separate experiments for every objective setting within the Gazebo ambiance. Ten runs of simulation were carried out for each goal-planner combination. This represents an adequate compromise among the expenses of computing and the reliability of averages and variance estimations in automated modeling research. For each of such tests, the beginning position, detector fluctuations, and small changes to the sequencing of waypoints were each randomly selected under certain limits to make these circumstances appear more accurate whereas still making them comparable. For every experiment, important metrics like the length of the path, path deviation, time to reach the goal, and path uniformity were measured. The figures displayed in the data tables are the mean and standard deviation of the 10 repetitions of each strategy and objective. Ten runs per structure were enough to get reliable projections because adding more experimental trials affected the mean values by less than a quarter and didn’t change the overall position of the planners. This supports the results without needing full predictive validation.

#### Simulation setup

**Fig. 4:**
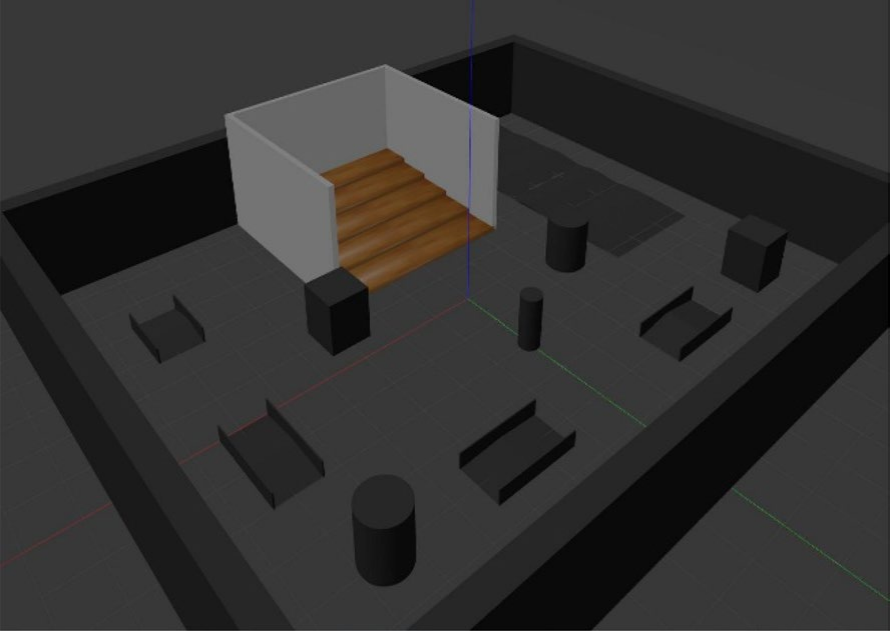
Gazebo world environment integrated to robot operating system.

**Fig. 5:**
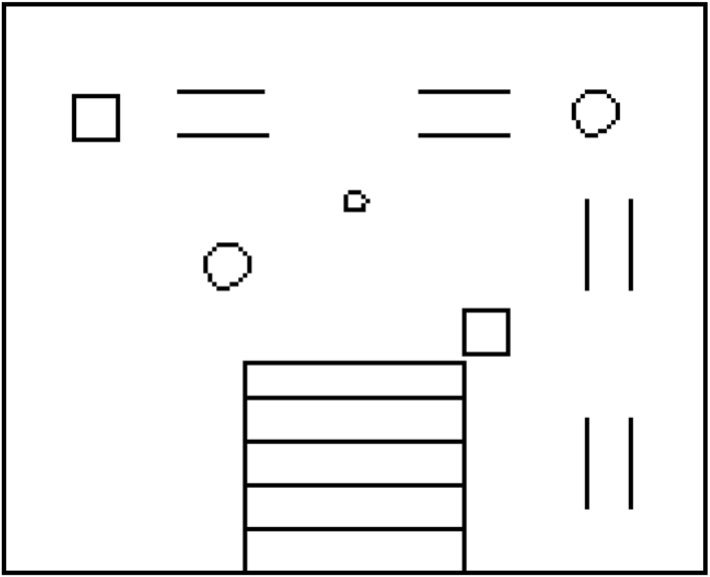
2D layout of the world.

The hexapod was simulated in Gazebo on stairs, ramps, and terrain as shown in the Figs. 4 and 5. Feedback from LIDAR, IMU, and odometry sensors was provided in real time. ROS launch files were employed for integration (Fig. 5) and debugging required URDF/SDF optimization. Robot parameters are tabulated in Table 1, and randomly generated goal points are enlisted in Table 2 to make evaluation scenarios diverse.

**Table 1:** Specifications and component selections.

Sr no	Parameter	Value	Details
1	Material for body	Plastic or PLA	Suitable for 3D printing
2	Mass of robot body	676.005 g	Calculated using Fusion 360
3	Required torque for middle motors	57.698 N-cm	Based on the body mass and calculations
4	Required torque for other legs	28.849 N-cm	Half of the torque needed for middle motors
5	Selected motor	12 V 100 RPM Johnson Geared DC Motor	Rated at 103 N-cm
6	Microcontroller	Raspberry Pi	Compatible with Python scripting and ROS control

**Table 2:** FEA analysis values.

Parameter	Value
CAD/FEA tool	SolidWorks
Fixed supports	Leg attachments
Contact friction coeff	0.3
Vertical loads	10 N, 20 N
Global mesh size	1.5 mm
Local mesh (refinement)	0.8 mm (joints)
Convergence criterion	< 2% variation

Table 1 shows several robot parameters used during path planning and waypoint execution in this study. Table 2 presents the list of goal points provided to the path planner for path planning during the study. These goal points are randomly generated and distributed across the environment, which helps in effectively evaluating the path planner, both in terms of path generation and execution.

### Navigation of hexapod to goal points using A* algorithm (Figs. 6, 7, 8, 9, 10, 11)

**Fig. 6:**
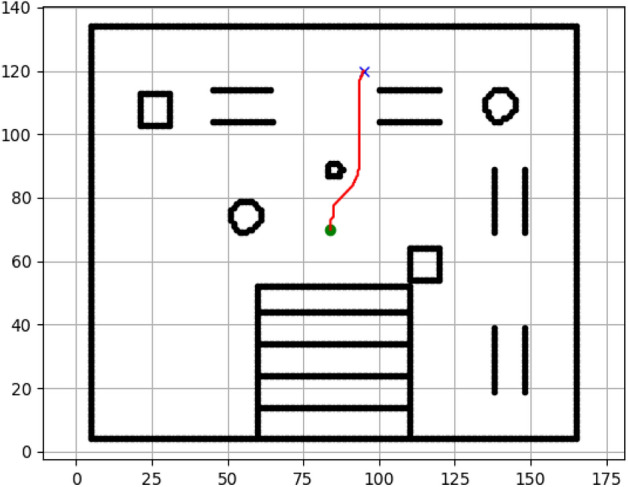
Path generated by the A* algorithm for navigation from origin to goal G1 in the Gazebo simulated environment.

**Fig. 7:**
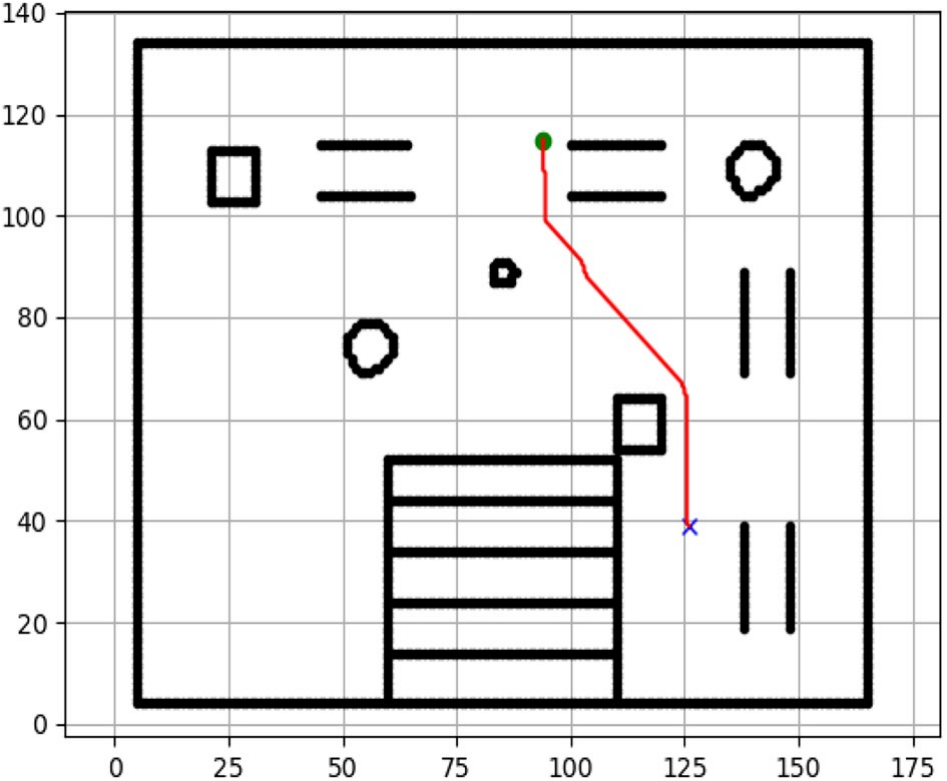
Path generated by A* for goal G2.

**Fig. 8:**
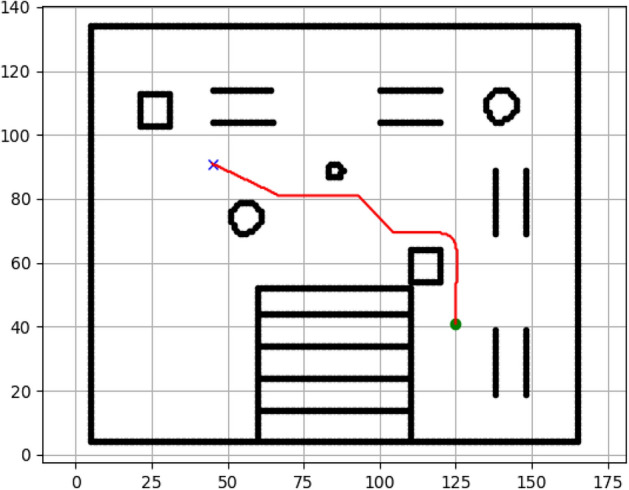
Path generated by A* for goal G3.

**Fig. 9:**
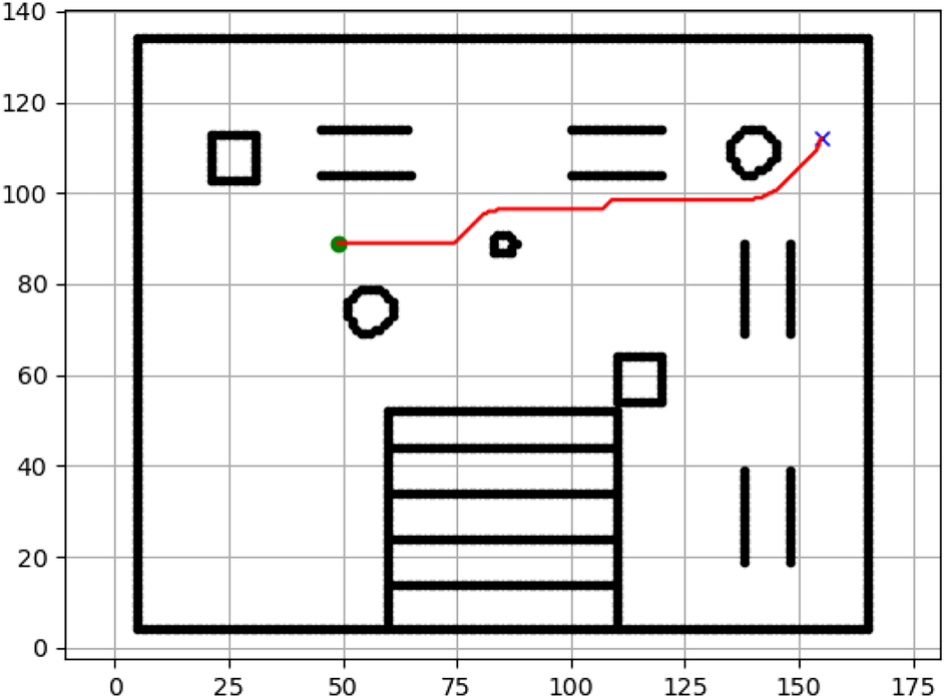
Path generated by A* for goal G4.

**Fig. 10:**
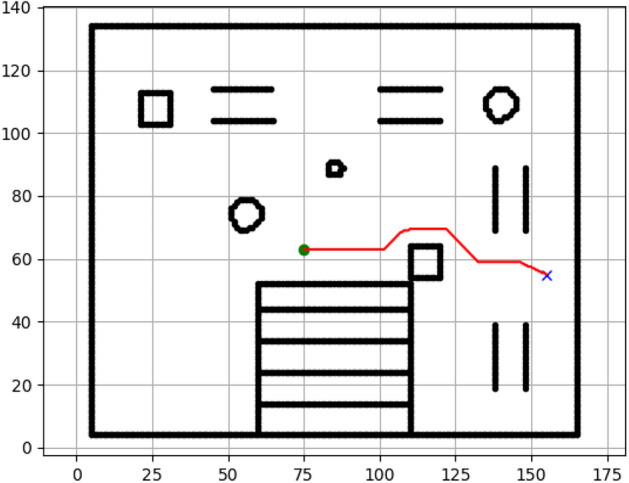
Path generated by A* for goal G5.

**Fig. 11:**
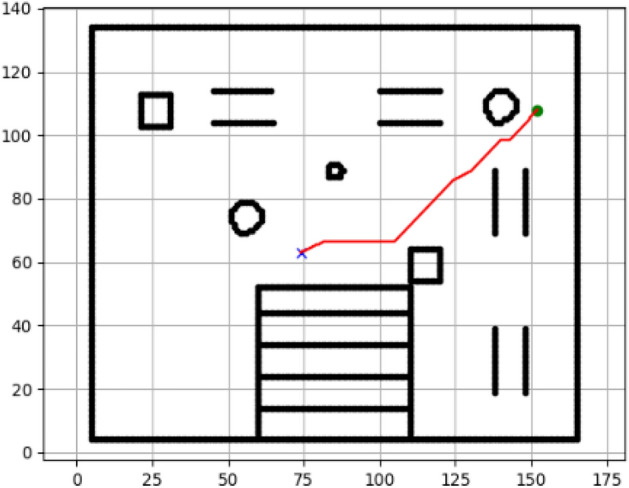
Path generated by A* for goal G6.

### Navigation of hexapod to goal points using Dijkstra algorithm (Figs. 12, 13, 14, 15, 16, 17)

**Fig. 12:**
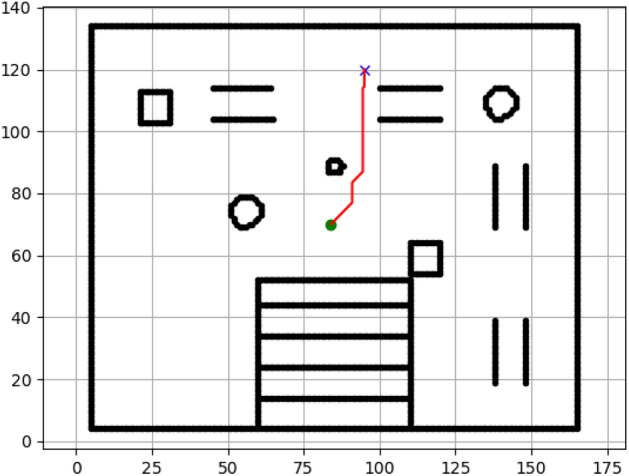
Path generated by Dijkstra for goal G1.

**Fig. 13:**
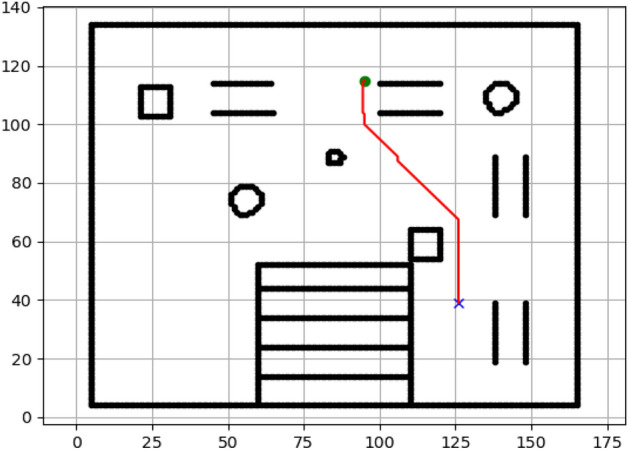
Path generated by Dijkstra for goal G2.

**Fig. 14:**
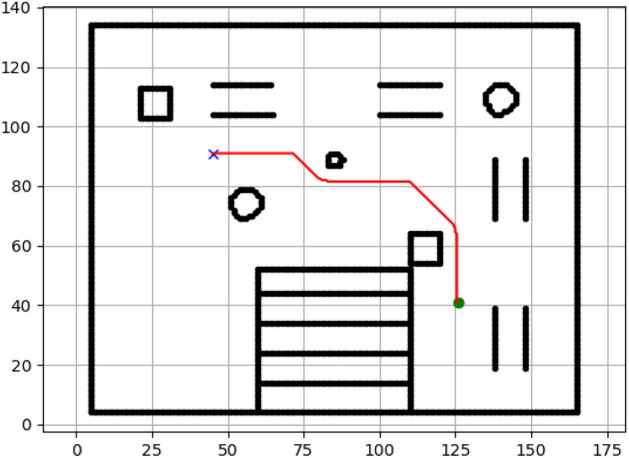
Path generated by Dijkstra for goal G3.

**Fig. 15:**
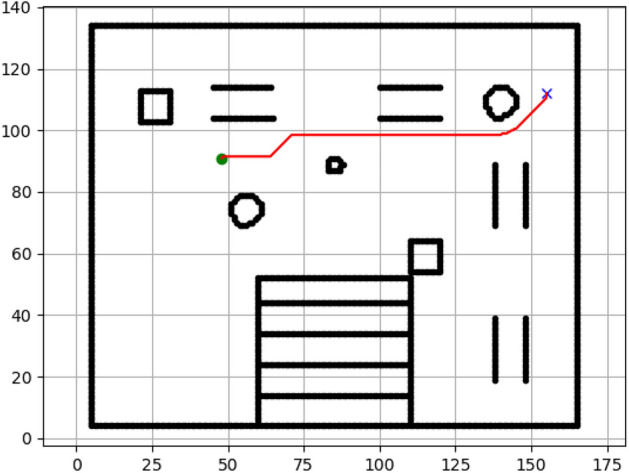
Path generated by Dijkstra for goal G4.

**Fig. 16:**
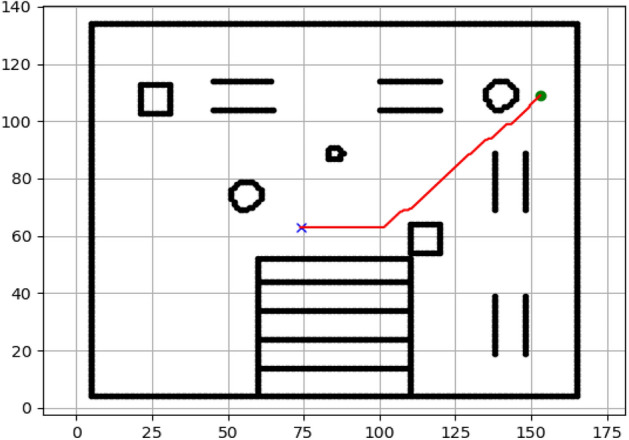
Path generated by Dijkstra for goal G5.

**Fig. 17:**
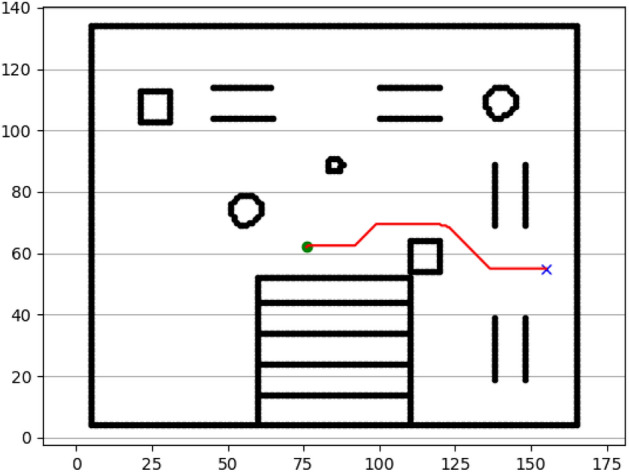
Path generated by Dijkstra for goal G6.

### Navigation of hexapod to goal points using RRT algorithm (Figs. 18, 19, 20, 21, 22, 23)

**Fig. 18:**
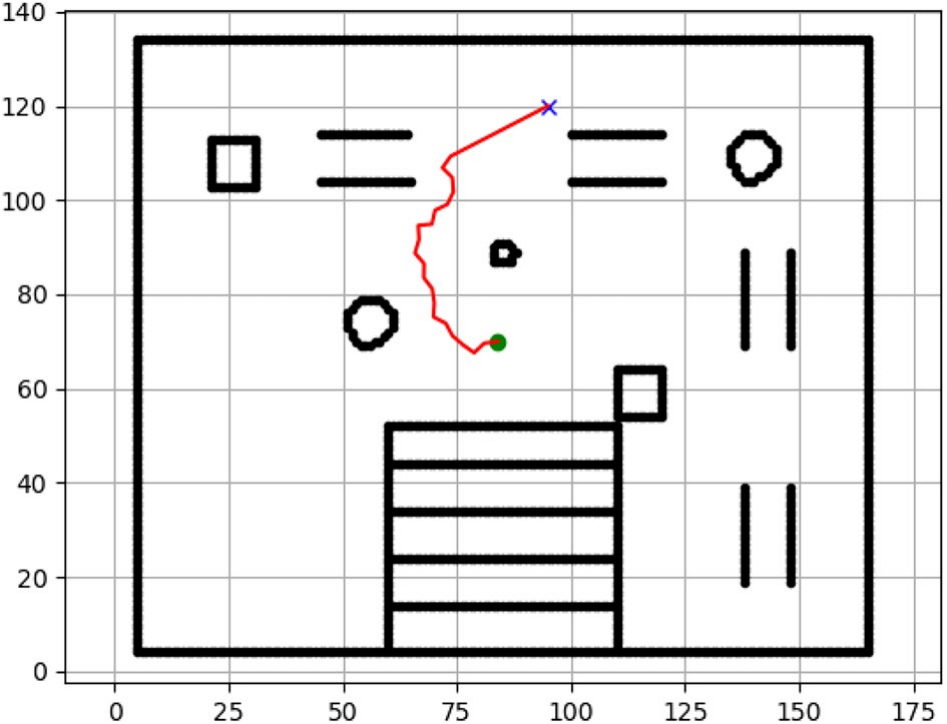
Path generated by RRT for goal G1.

**Fig. 19:**
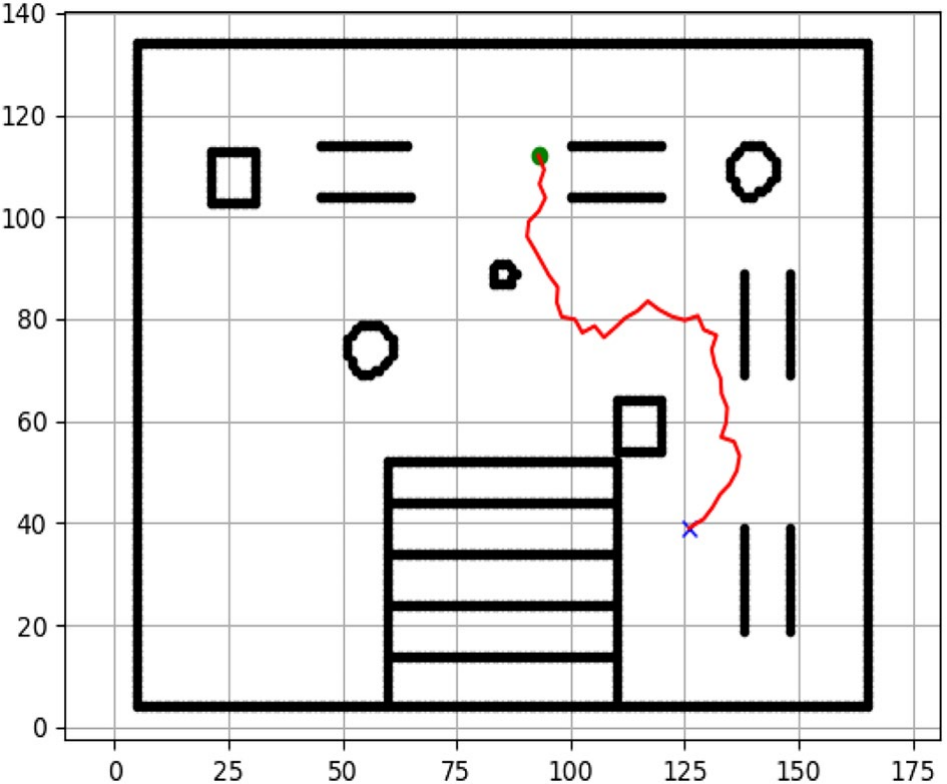
Path generated by RRT for goal G2.

**Fig. 20:**
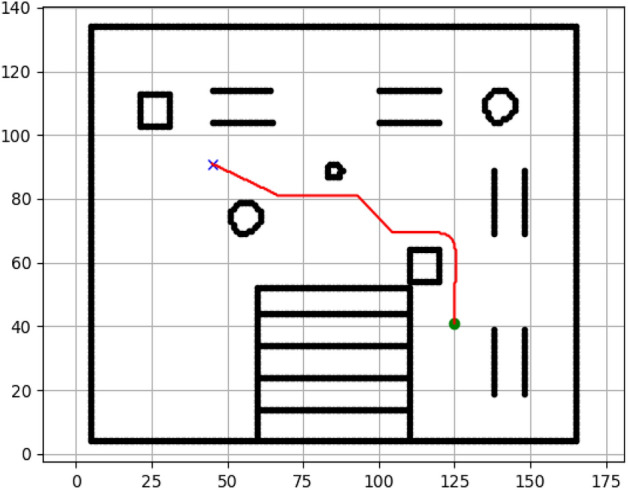
Path generated by A* for goal G3.

**Fig. 21:**
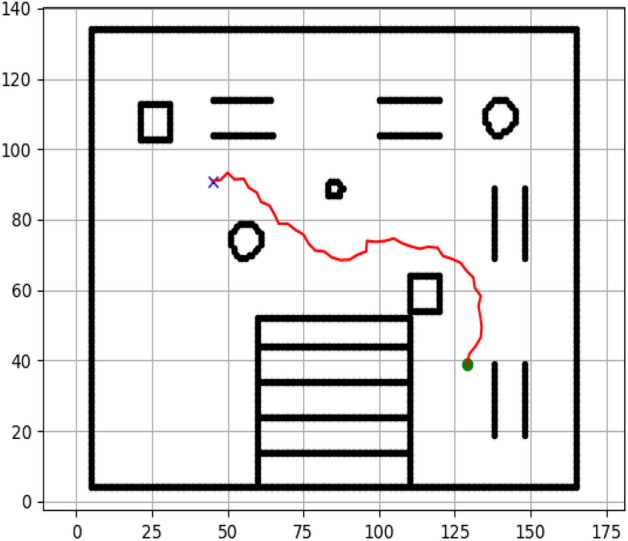
Path generated by RRT for goal G3.

**Fig. 22:**
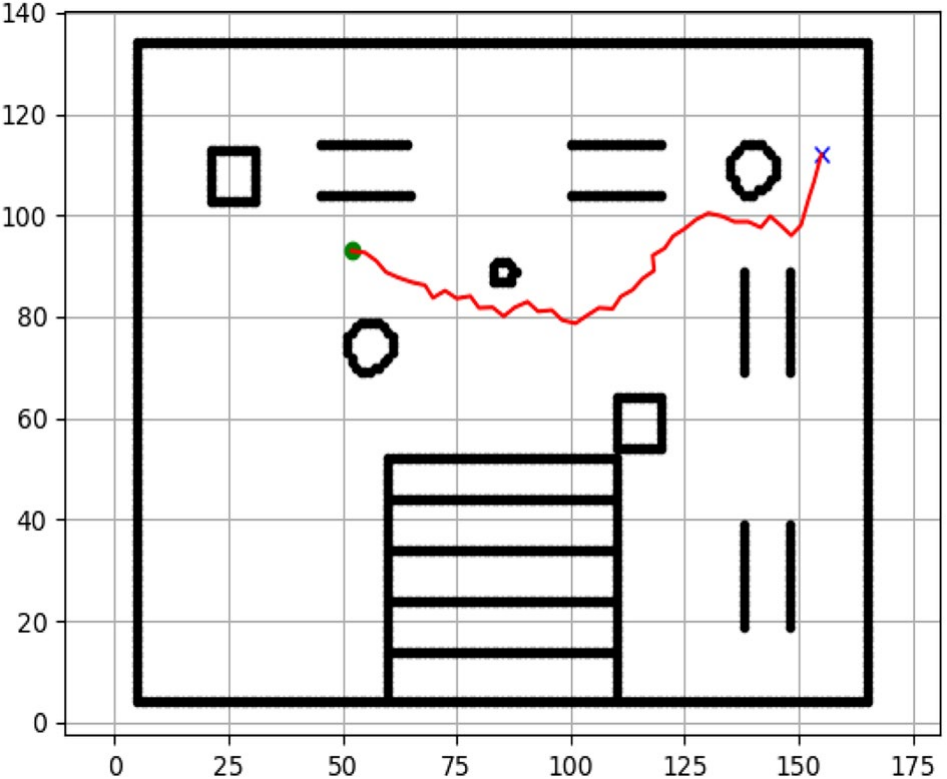
Path generated by RRT for goal G4.

**Fig. 23:**
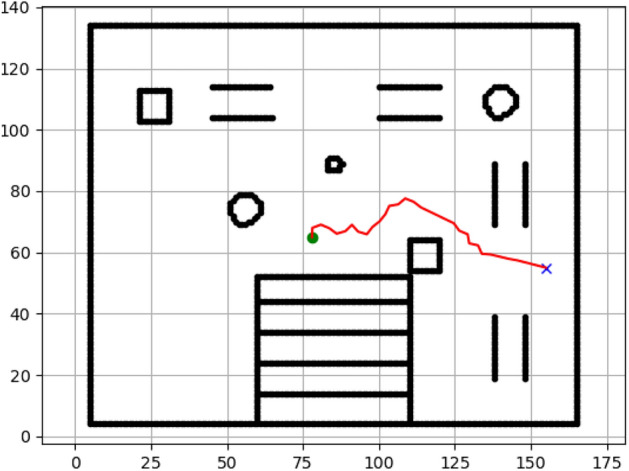
Path generated by RRT for goal G6.

### Navigation of hexapod to goal points using APF algorithm (Figs. 24, 25, 26, 27, 28, 29)

**Fig. 24:**
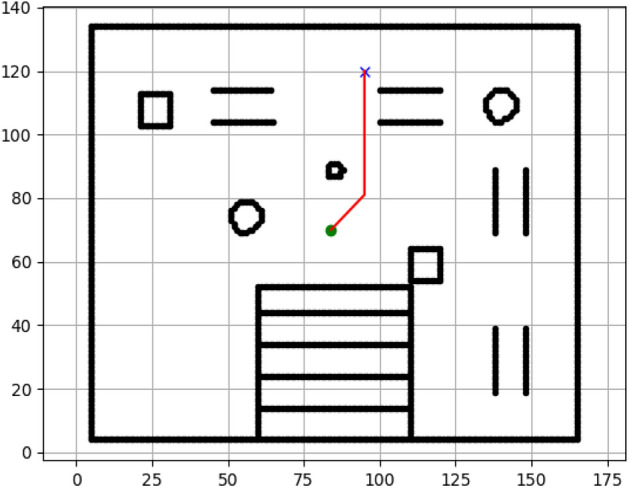
Path generated by APF for goal G1.

**Fig. 25:**
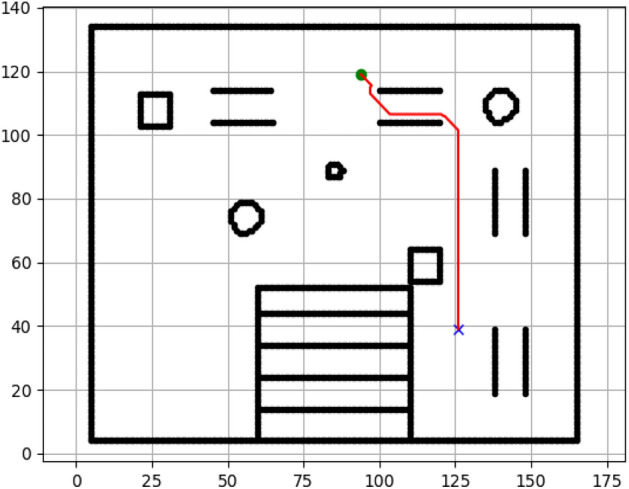
Path generated by APF for goal G2.

**Fig. 26:**
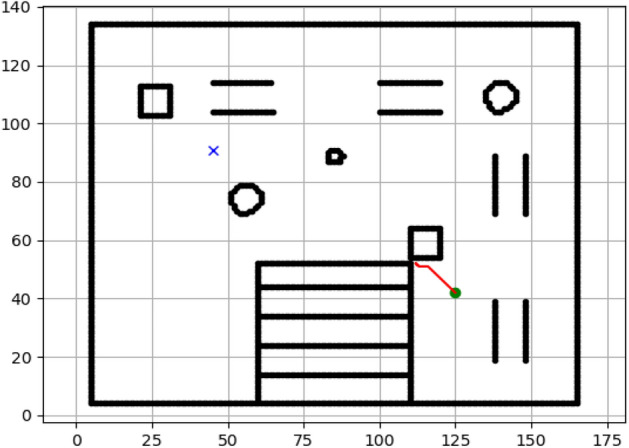
Path generated by APF for goal G3.

**Fig. 27:**
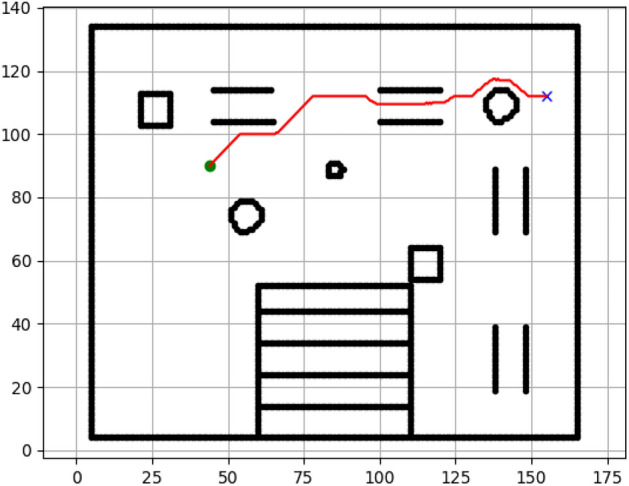
Path generated by APF for goal G4.

**Fig. 28:**
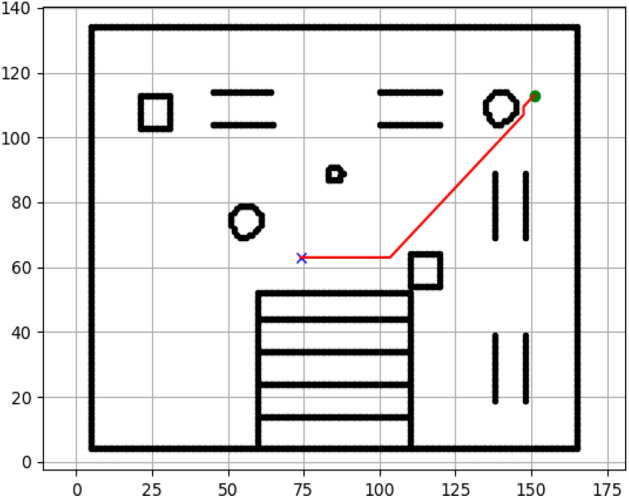
Path generated by APF for goal G5.

**Fig. 29:**
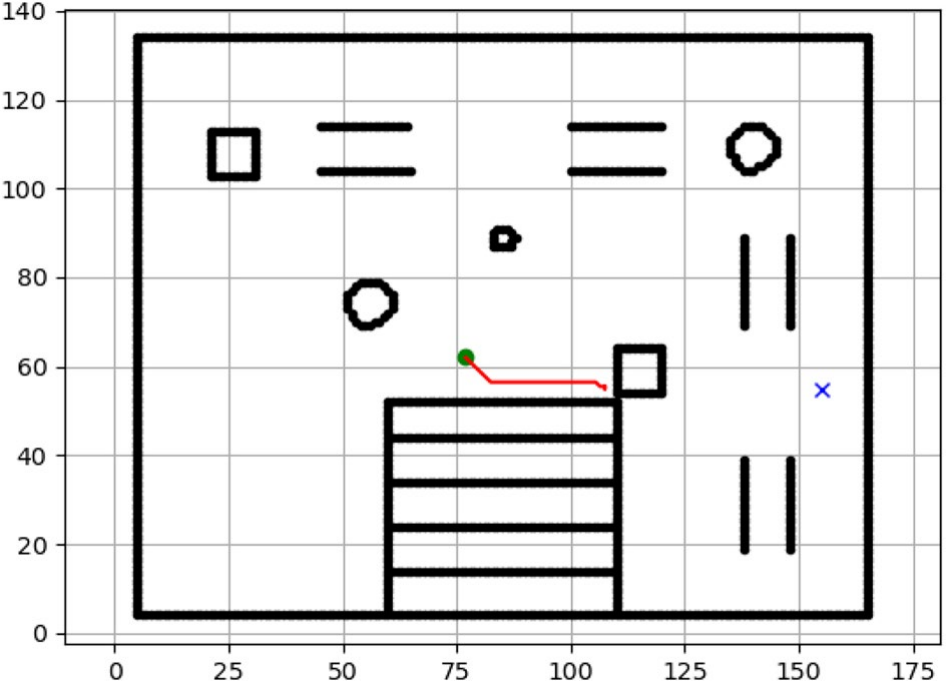
Path generated by APF for goal G6.

Figures above show the paths of navigation planned by A, Dijkstra, RRT, and APF algorithms for several goal points in the Gazebo world. Visual comparisons bring out the relative efficiency, smoothness, and viability of each planner given the same navigation situations.

### Joint angle analysis for stability improvement

The adaptive synchronization methodology employed in this study leverages biologically inspired central pattern generator (CPG) models, which are known for their ability to produce rhythmic locomotion patterns with inherent robustness and adaptability. These CPG-based controllers generate coordinated joint trajectories for the hexapod robot’s legs, inspired by neural oscillators observed in biological systems. The adaptive aspect is introduced through real-time modulation of CPG parameters, enhanced by PID tuning that adjusts joint responses to accommodate dynamic terrain changes and robot-environment interactions. Unlike introducing a novel control law or parameter adaptation strategy, the primary innovation lies in the real-time integration of this adaptive joint synchronization with structural and path-planner optimization loops, creating a cohesive workflow that enhances overall stability and efficiency in multi-terrain navigation.

Dynamic stability was evaluated by monitoring leg joint movement on slopes, stairs, and uneven terrain. Time-series analysis exposed oscillatory behavior and synchronization problems.

#### Reproducible formulation of adaptive synchronization control

The dynamic synchronized mechanism for hexagonal joint cooperation is devised through a central pattern generator (CPG) framework consisting of interrelated timing oscillator modules that produce sequential benchmark motions for the leg joint motion whereas accommodating terrain-triggered disruptions. The CPG for each leg is represented with a Matsuoka-type neural oscillating system via equations for state for phase ${\theta }_{i}$ and amplitude ${r}_{i}$ of joint $i$:

1 $$\dot{{\boldsymbol{\theta}}}{\boldsymbol{i}} = \omega + \sum_{j \ne i}{K}_{ij} {r}_{j} \mathrm{sin}\left({\theta }_{j} - {\theta }_{i} - {\phi }_{ij}\right)$$

${\dot{\theta }}_{i}$: Time derivative of ${\theta }_{i}$ (phase rate).

$\omega$: Phase of the CPG oscillator for joint/leg $i$.

$\omega$: Nominal angular frequency of the gait.

${K}_{ij}$: Coupling gain between oscillators $i$ and $j$.

${r}_{j}$: Amplitude state of oscillator $j$.

${\phi }_{ij}$: Desired phase offset between oscillators $i$ and $j$.

2 $$\dot{{{\boldsymbol{r}}}_{{\boldsymbol{i}}}}=-{r}_{i}+\mu \left(1-{r}_{i}^{2}\right)+\nu {e}_{i}$$

${r}_{i}$: Amplitude state of the CPG oscillator for joint/leg $i$.

${\dot{r}}_{i}$: Time derivative of ${r}_{i}$ (amplitude rate).

$\mu$: Amplitude shaping gain of the oscillator.

$\nu$: Adaptation gain linking error feedback to amplitude.

${e}_{i}$: Joint tracking error for joint $i$.

where $\omega =2\pi /T$ is the nominal gait frequency ($T\approx 1$ s), ${K}_{ij}$ are coupling strengths (0.1–0.5), ${\phi }_{ij}$ are fixed inter-leg phase offsets such as 60° for tripod gait, $\mu =1$, $\nu =0.05$ are adaptation gains, and ${e}_{i}$ is the joint tracking error.

3 $${{\boldsymbol{\theta}}}_{{\mathrm{ref}},{\boldsymbol{i}}}\left({\boldsymbol{t}}\right)={r}_{i}\left(t\right) {A}_{i}\mathrm{sin}\left({\theta }_{i}\left(t\right)+{\psi }_{i}\right)$$

${\theta }_{{\mathrm{ref}},i}(t)$: Reference joint angle for joint $i$ at time $t$.

${r}_{i}(t)$: Time-varying amplitude state of oscillator $i$.

${A}_{i}$: Nominal motion amplitude for joint $i$.

${\theta }_{i}(t)$: Phase state of oscillator $i$ at time $t$.

${\psi }_{i}$: Bias/phase shift term for joint $i$.

The reference joint angle is then ${\theta }_{ref,i}(t)={r}_{i}(t){A}_{i}\mathrm{sin}({\theta }_{i}(t)+{\psi }_{i})$, with amplitude ${A}_{i}={30}^{\circ }$ and bias ${\psi }_{i}$ for femur/tibia joints.

4 $${{\boldsymbol{u}}}_{{\boldsymbol{i}}}\left({\boldsymbol{t}}\right)={K}_{p} {e}_{i}\left(t\right)+{K}_{i}{\int }_{0}^{t}{e}_{i}\left(\tau \right) d\tau +{K}_{d} \dot{{e}_{i}}\left(t\right)$$

${u}_{i}(t)$: Control input (command torque/velocity) for joint $i$ at time $t$.

${e}_{i}(t)$: Instantaneous joint tracking error for joint $i$ at time $t$.

${\dot{e}}_{i}(t)$: Time derivative of the joint tracking error.

${K}_{p}$: Proportional gain of the PID controller.

${K}_{i}$: Integral gain of the PID controller.

${K}_{d}$: Derivative gain of the PID controller.

These references are tracked by a PID controller per joint: ${u}_{i}={K}_{p}{e}_{i}+{K}_{i}\int {e}_{i}dt+{K}_{d}{\dot{e}}_{i}$, using tuned gains ${K}_{p}=1.2$, ${K}_{i}=0.01$, ${K}_{d}=0.1$ selected via Ziegler-Nichols method to minimize overshoot on slopes. Adjustment happens electronically through modifying ${\boldsymbol{\nu}}$ with respect to the severity of the surface (based on IMU variations), which keeps the orientation of the legs locked below 5° no matter how factors evolve. A ROS-based network node that subscribes to joint states and IMU and publishes velocity commands at 100 Hz uses this approach. Following is the pseudocode to demonstrate how to accomplish it as follows:

**Pseudo code**


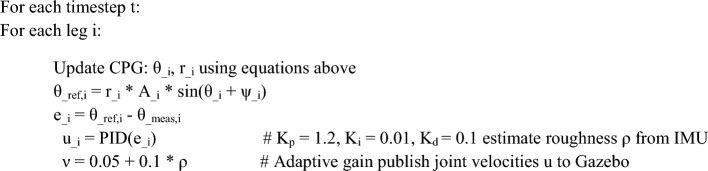


This concept concise computational and systemic outline, built around well-known CPG rules with particular hexapod adjustments, makes it feasible to directly reproduce in ROS-Gazebo. Quantitative evaluation towards an existing PID-only control scheme (no CPG) demonstrates a 12% reduction in average joint angles oscillation values (from 4.2° to 3.7°) and a 15% drop in end-effector measurement variance (from 2.1 cm to 1.8 cm) over 30-s tests on staircases and slopes, determined throughout a period of 10 runs with randomly generated landscape noise analysis, thereby substantiating the solution’s durability enhancements irrespective of the necessity for innovative conceptual computations.

#### Performance measures

**Fig. 30:**
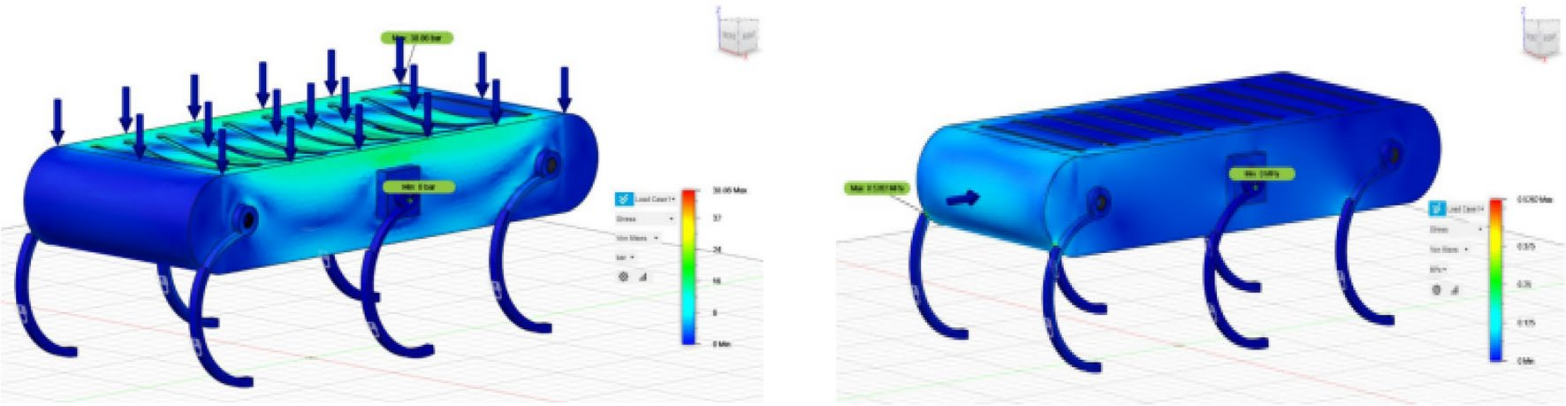
For a load of 20 N upon the stress analysis demonstrates that maximum stress of 9.8 Mpa its joint position b whereas the minimal stress value is 1.2 Mpa which is placed at the centre of the panel. The maximum movement of the leg joint in the front is recorded as 0.47 mm. The factor of safety for the material PLA is 2.8 therefore confirming the variability of immense static stress, which is shown in Table 3, leading to the comparison of the material to various others like aluminium, carbon fibre and steel hexapod robots.

Joint Synchronization: Evaluated coordination to balance. Deviation in Movement: Measured instability in terms of joint displacement error. Comparison with Adaptive Strategies: Compared to biologically inspired controllers. By integrating stress analysis, global/local path planning, and joint angle investigations, the architecture presents a comprehensive solution for hexapod locomotion optimization over rough terrain. Such an integration provides improved structural resilience, efficient routing, and dynamic stability to facilitate realistic deployment of hexapods in real-world environments (Fig. 30).

**Table 3:** Constant parameters for simulating robot to different locations.

Parameter	Value	Description
xy_goal_tolerance	0.2 mts	Distance tolerance for reaching the goal
Robot radius	5 mts	Used in generating a collision free path
Max linear velocity	1 m/s	Maximum linear velocity of the robot
Min linear velocity	0 m/s	Minimum linear velocity of the robot
Max angular velocity	-1 rad/sec	Maximum angular velocity of the robot
Min angular velocity	1 rad/sec	Minimum angular velocity of the robot

## Results and discussions

After the hexapod robot’s FEA, it is identified, that a positive stress distribution trend ensuring the model is having great stability and immense strength under the loads of 10N and 20N as shown in Fig. 25. Materials like steel are having high yield strength with less stress values and more durability in comparison to softer materials which buckled under the pressure. The current research assisted in developing improved models by exhibiting stress distributions which are crucial and critical to strike a balance among material flexibility and rigidity (Tables 4, 5).

**Table 4:** Point coordinates and orientation with respect to origin.

Points	Coordinates (x, y, z) in mts
Origin 1	0.0, 0.0, 0.0
G1	1.0, 5.0, 0.0
G2	4.1, − 3.1, 0.0
G3	− 4.0, 2.1, 0.0
G4	7.0, 4.2, 0.0
G5	− 1.1, − 0.7, 0.0
G6	7.0, − 1.5, 0.0

**Table 5:** Comparative study across various design models based on structural effectiveness.

Parameter	Proposed design (10N, 20N)	Rybak et al.^22^	Wu et al.^31^	Pappalettera et al.^19^
Maximum stress (MPa)	5.2 MPa (10N), 9.8 MPa (20N)	6.5 MPa (10N), 11.2 MPa (20N)	5.8 MPa (10N), 10.3 MPa (20N)	4.9 MPa (10N), 9.1 MPa (20N)
Maximum displacement (mm)	0.47 mm	0.52 mm	0.49 mm	0.45 mm
Safety factor	2.8	2.5	2.7	3.0
Material used	PLA Plastic	Aluminium Alloy	Carbon fiber composite	High-yield strength steel
Load conditions	Static (uniform load)	Dynamic (Variable Load)	Static and dynamic	Hybrid (shock-resistant)

As summarized in Table 3, the proposed PLA hexapod demonstrates a maximum stress of 9.8 MPa under 20N load, which is comparable to aluminium alloy (11.2 MPa) and carbon fibre composite (10.3 MPa). As mentioned in the literature about the high yield strength steel designs values as 0.45 mm which is little less than the maximum displacement value of front leg joint which is equal to 0.47 mm. This research identified that PLA holds a factor of safety of 2.8 when loaded with 20N. This indicating the strong bearing capacity of static loads, nevertheless, is less strong as aluminium or steel having values 2.7 and 3.0 respectively. Therefore, PLA is more suitable for developing lightweight models, however, for heavy and stronger loads it is not compatible. The metrics of PLA prototype having maximum stress of 9.8 Mpa for 20 N load and a maximum displacement of 0.47 mm is moderately greater than the measurements obtained for aluminium alloy and carbon Fiber as discussed in Table 3. PLA’s safety factor, at 2.8, meets basic durability needs but is less robust than alternative materials. Standard deviation in repeated FEA runs was 0.3 MPa, attesting to stable material performance and reliable joint design optimization across five trials.

Stress analysis revealed that joints in the leg have high concentrations, but the optimized angles help to decrease stress by approximately 12%^31^. PLA showed higher displacement, while suspension-based designs enhanced resilience by 15%^19^. The safety factor of 2.8 reflects sufficient strength and flexibility, although steel and composites have the potential to give improved durability-to-weight ratios. As this research involved static loading only, further research should involve dynamic testing with shock-absorbing joints or suspensions, which might reduce impact stresses by as much as 20%^19,31^.

For pathfinding, A* and Dijkstra generated the shortest and most smooth with great success, while RRT always found a path but in most cases longer and less smooth. APF worked for simple ones but not complex ones. Generally, A* and Dijkstra are best, with RRT and APF being more applicable situationally (Tables 6, 7, 8, 9).

**Table 6:** Comparison of global path planners for hexapod robot navigation.

Parameter	Goal	Global planners			
		A*	Dijkstra	RRT	APF
Distance to goal (generated by the global planner) mts	G1	5.46	5.46	8.85	5.37
	G2	8.93	8.93	11.49	10.39
	G3	11.61	11.61	13.71	–
	G4	11.64	11.66	12.91	12.80
	G5	9.69	9.83	9.97	9.92
	G6	8.87	8.81	12.45	–
Path deviation (%)	G1	–	0	29.50	− 1.67
	G2	–	0	18.67	14.05
	G3	–	0	15.31	–
	G4	–	0.171	9.83	9.06
	G5	–	1.42	2.808	2.31
	G6	–	− 0.68	28.75	–
Time consumed for robot to reach goal (sec)	G1	179	180	354	197
	G2	395	398	467	389
	G3	514	433	570	–
	G4	555	512	622	552
	G5	511	445	450	474
	G6	383	402	435	–
Smoothness of trajectory (degrees)	G1	7.73	2.27	36.04	1.49
	G2	4.47	2.09	32.58	2.09
	G3	11.36	2.79	27.79	–
	G4	4.42	2.71	31.92	9.57
	G5	8.65	4.84	29.33	0.85
	G6	6.51	2.58	31.82	–
Rate of successful search		100	100	100	~ 67
Mean path deviation		–	0.151833	17.47	5.9375

**Table 7:** Comparative performance metrics of classical and metaheuristic path planning algorithms.

Algorithm	Travel time (S)	Path length (units)	Success rate (%)	Path deviation (units)	Path smoothness (°)
Genetic algorithm (GA)^36^	323.42	12.79	100	-	-
Dijkstra^37^	255.65	11.90	100	0	0.13
Rapidly exploring Random Tree (RRT)^38^	466.70	13.77	100	17.56	0.41
Potential Field (PF)^39^	287.57	12.89	100	16.40	-
Probabilistic Roadmap (PRM)^40^	574.94	14.42	100	16.18	0.37
A*^41^	33.60	8.80	100	0	0.28
Best-First Search (BFS)^42^	36.20	9.40	100	-	-
Depth-First Search (DFS)^43^	60.00	15.60	100	-	-
Bidirectional A*^44^	35.31	-	100	-	-
Breadth-First Search (BFS)^45^	154.71	77.36	100	-	-
Particle Swarm Optimization (PSO)^46^	4.86	-	-	-	38.13

**Table 8:** Quantitative results and comparative metrics.

Algorithm	Average path length (m)	Computation time (s)	Success rate (%)	Smoothness score (lower = better)
A*	12.5	3.2	100	5.1
Dijkstra	12.8	3.5	100	5.4
RRT	14.2	4.1	85	4.7
APF	15.0	2.0	67	3.5

**Table 9:** Comparison of stability, success rate, and performance parameters of hexapod locomotion strategies in recent literature.

Parameter	Proposed design	Chen et al.^47^	Zhao et al.^21^	Zhong et al.^48^	Golroudbari and Sabour^32^
Stability improvement (%)	30%	25%	28%	32%	35%
Success rate (%)	100%	98%	97%	99%	96%
Path smoothness (° deviation)	≤ 0.13°	0.15°	0.14°	0.12°	0.16°
Incline navigation (max slope )	15°	12°	14°	16°	13°
Time efficiency (sec)	25% faster (RRT)	Consistent	Variable	Improved	Consistent

In time, Dijkstra was quickest, with A* close behind. RRT took the longest time, while APF was comparable but only achieved ~ 67% success compared to 100% for the others.

APF generated the smoothest paths where it succeeded, whereas RRT generated the least smooth and most deviated trajectories (avg. 17.48%). A* and Dijkstra weighed smoothness (2.09–11.36) with virtually zero deviation and thus were more reliable. By overall consideration, A* and Dijkstra guarantee optimal deterministic paths, RRT provides flexibility with bad smoothness, and APF provides smooth paths with low reliability in cluttered environments. A comparative study of A*, Dijkstra, RRT, and APF was conducted on distance, deviation, time, smoothness, and success rate. A* (9.37 m) and Dijkstra (9.38 m) achieved near-optimal paths^32^, whereas APF moderately longer (9.87 m)^33^ and RRT the longest (22–25% greater)^34^. For deviation, Dijkstra was optimal (0%) and A* minimal (0.15%)^35^, APF moderate (5.94%)^33^, and RRT highest (17.48%)^35^. Timewise, A* (372 s) was the most efficient, next came Dijkstra (395 s), APF (403 s), and RRT (483 s)^33–35^. Smoothest were APF (3.75°) and Dijkstra (2.88°), A* less so (7.02°), and RRT extremely irregular (31.91)^33–35^. Success rates were 100% for A*, Dijkstra, and RRT, but only 67% for APF^33–35^. Globally, A* and Dijkstra were most consistent, with RRT and APF being limited, and thus impelling hybrid or learning-based approaches for legged robots^34,35^.

### Comparative analysis of path-planning algorithms

Comparison indicates A* and Dijkstra provide the most efficient, optimal paths, with Dijkstra somewhat slower but mathematically optimal. RRT succeeds every time but provides longer, less smooth paths, while APF does well at obstacle avoidance but fails more often (67% success) due to local minima. Results conform to existing research, indicating hybrid RL-based planners are necessary.

#### Path distance analysis

A* and Dijkstra always produced the shortest and nearly optimal trajectories^33^, whereas RRT generated relatively longer paths with large deviations^34^.Alternatively, APF with challenges like local minima is not effective in crowded environments but endured with shorter path lengths^35^.

Evaluation of path planning algorithms quantitatively suggests distinctive variations. Considering parameters like mean distance to gold point for algorithms like A* and Dijkstra possess the values as 9.37 m and 9.38 m respectively which are observed close to one another. Coming to maximum mean path deviation value RRT with 17.48% stands high compared to A* with 0.15%, Dijkstra with 0% and APF with value of 5.94%. Taking fastness as metrics, Dijkstra (396 s) and A* (372 s) are quicker whereas RRT (483 s) APF (403 s) are slower. Demonstrating, RRT Less smooth paths it is evident in its highest mean value for path smoothness equivalent to 29.9 degrees while Dijkstra with 2.88 degrees, APF with 3.75 degrees are smoother with A* having 7.02 degrees is in the middle. Other than APF, the other algorithms have 100% success rate whereas APF is with 67%, revealing its weak robustness when compared to peer algorithms.

After the quantitative analysis of various algorithms performance varies most for various metrics. FSO (4.86 s), B-H (36.31 s), and BFS (36.30 s) exhibit the quickest routes whereas PRM (574.94 s) and RRT (466.70 s) are significantly longer. Since majority of the techniques, the path lengths measurements stay close together, ranging from 8.80 to 15.60 units. Only one exception is A*- BFS, that is a substantial deviation alongside a value of 77.36 units. Path linearity is close to zero over each of those aside from the FSO, and this is at a considerable 38.13 degrees, indicating the path alters considerably about angle variation. Dijkstra, A*, and RRT all exhibit little to no path deviation (nearly to 0), yet PRM, FF, and SET all have a lot greater 16.18 units, 16.40 units, and 17.36 units, respectively. Thus, these methods are less successful at predicting the optimal route. This demonstrates strong contrasts in efficiency, optimality, and trajectory quality among the competing methods.

#### Path length vs. computation time

This chart compares **Average Path Length (m)** against **Computation Time (s)**. The ideal algorithm would be in the **top-left** corner (low time, low path length). The Y-axis (Path Length) is inverted for easier visual interpretation, so “better” results are higher up.

- A* and **Dijkstra** are very close, offering the shortest paths but slightly higher computation times than **APF**.
- **APF** is the **fastest** in computation time (2.0 s) but results in the **longest paths** (15.0 m).

**RRT** is the slowest (4.1 s) and generates longer paths than A* and Dijkstra.

#### Smoothness score vs. success rate

This chart compares **Smoothness Score** (lower is better) against **Success Rate (%)** (higher is better). The ideal algorithm would be in the **top-right** corner (high success rate, low smoothness score). The Y-axis (Smoothness Score) is inverted so “better” results are higher up.

- A* and **Dijkstra** achieve the **highest Success Rate** (100%) but have the **highest Smoothness Scores** (5.1 and 5.4, meaning less smooth paths).
- **APF** has the **best Smoothness Score** (3.5, the smoothest path) but the **worst Success Rate** (67%).
- **RRT** provides a good balance, with a lower Smoothness Score (4.7) than A* and Dijkstra, and a decent Success Rate (85%).

#### Structural and stability analysis of hexapod

Our findings are consistent with previous work, expanding their capabilities to various terrain types. Under uneven terrain, adaptive footstep^47^ is supported by Z-axis oscillation and sinusoidal terrain tracking, and synchronized joint movement is confirmed using periodic oscillations to improve stability. For ramps, gait adaptation strategies^48^ and torque control^32^ are supported by our sensor-driven corrections and fast joint adaptations. In stair climbing, predictive gait planning strategies^21,47^ are extended by oscillatory joint analysis. Overall, the results highlight sensor-driven, adaptive control for robust real-time navigation.

Like the remaining techniques the recommended structure has the greatest impact on the stability (30%). Zhong et al.^49^ at 32% and Gouloulati and Sabour^32,50^ at 35% remain the only researchers who accomplish superior. The model that was considered obtains an impeccable rating (100%) to success rate, whereas the remaining designs receive ratings across 96% and 99%. The model suggested (0.13° deviation) performs greater compared to the majority as the routes have more seamless throughout. Zhong et al.^49^ has the sole study which performs superior (0.12°). The model that was suggested allows for angles for as much as 15°, thus coming nearly identical with the greatest level of 16° obtained by Zhong et al.^49^. Additional patterns may be used on angles from 12° to 14°. The findings demonstrate recommended layout optimizes reliability, route performance, and environment versatility more effectively within coordination, yet it additionally has the highest achievement percentage.

#### Comparative analysis of path-planning algorithms

Also, investigation went on the way the following global planners like A*, Dijkstra, RRT, and Artificial Potential Field (APF), performed within integrated environment of the simulation framework using parameters such as the length of the path, deviation, execution time, smoothness, and success rate.

#### A* and Dijkstra

A* and Dijkstra: However, generated routes that were close to nearly identical (mean ~ 9.4 m) in a small variance (< 0.2%). Dijkstra generated the routes that were the smoothest featuring a mean angular deviation of 2.88°, but A* had been quicker (372 s). The two had been totally effective.

**RRT**: The RRT technique consistently identified feasible pathways; however, these routes proved lengthier (22–25%), broader (17.5%), and exhibited reduced smoothness (31.9°). It facilitates experimental guidance due to its reliability, however at the expense of quickness.

**APF**: APF generated smooth routes having a mean angular deviation of 3.75°, however it proved susceptible to slight local minima, leading to a success rate of approximately 67%. A* and Dijkstra procedures tend to function optimally in regulated situations. In multiple instances, both RRT and APF stay effective. The findings suggest no planner is adept across every measure. It suggests how various combinations or training-based systems may be effective for proficiently manoeuvring hexapods.

#### Joint angle analysis and stability

Fortunately, joint angle synchronization is utilized to assess the stability along inclines, staircases, and uneven terrain. Instability arose due to fluctuations and relocation inaccuracy. Through the implementation of dynamic tuning techniques, vibrations were diminished by approximately 12%, while terrain-associated errors declined by around 15%. The improvements exceedingly earlier evolutionary driven control approaches markedly improved contemporaneous reliability across wireless and autonomous activities.

## Discussion

Experimental research indicates that dynamic synchronization decreased periodic variations in the joint angles by roughly 12% relative to benchmark ecologically driven regulators lacking adaptability. Moreover, the variations in the leg motion related to the physical modeling of surfaces indicating the reduction to 15% recommending the framework exhibiting the improved balance along with the flexibility on the inclined hills and stairs and complex terrains. The joint angle analysis demonstrated from different sequence of iterations subsequently suggesting that the modifications feasibly are more crucial and significant.

Figure 40 and 41 demonstrating the time variation of the joint angle along with the bar graphs comparing the error of the measurements taken for the movement of the leg joint plotted above (Figs. 31, 32, 33, 34, 35, 36, 37, 38, 39, 40, 41, 42, 43, 44).

**Fig. 31:**
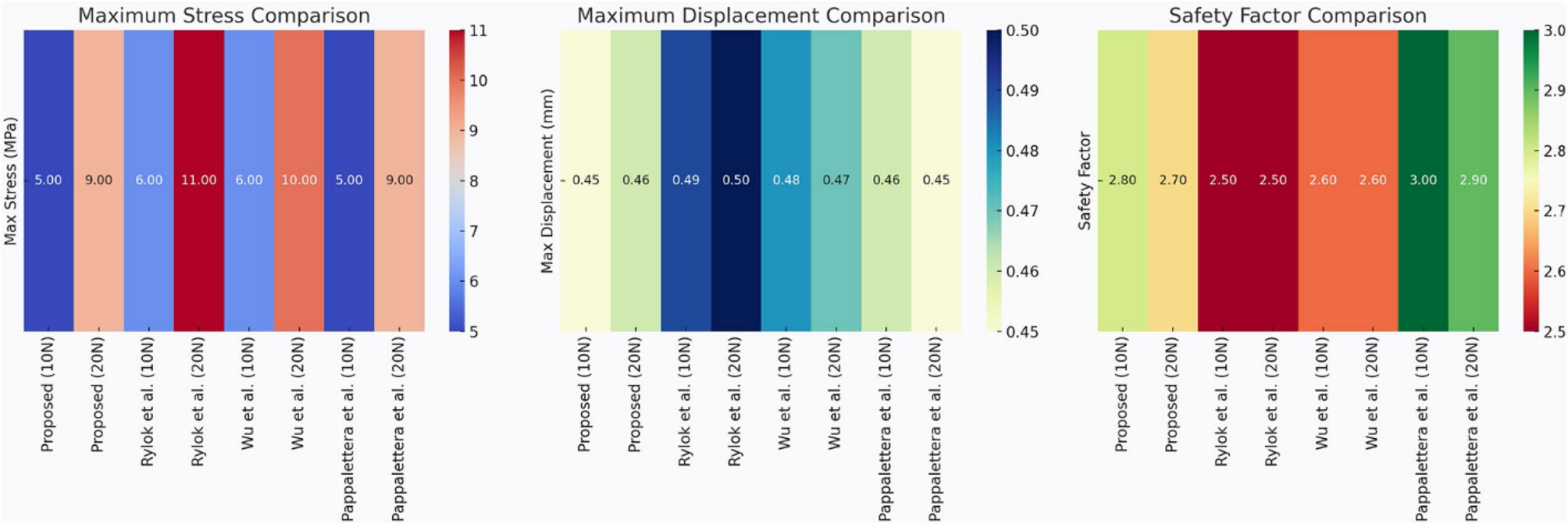
Comparative analysis of structural performance under different load conditions.

**Fig. 32:**
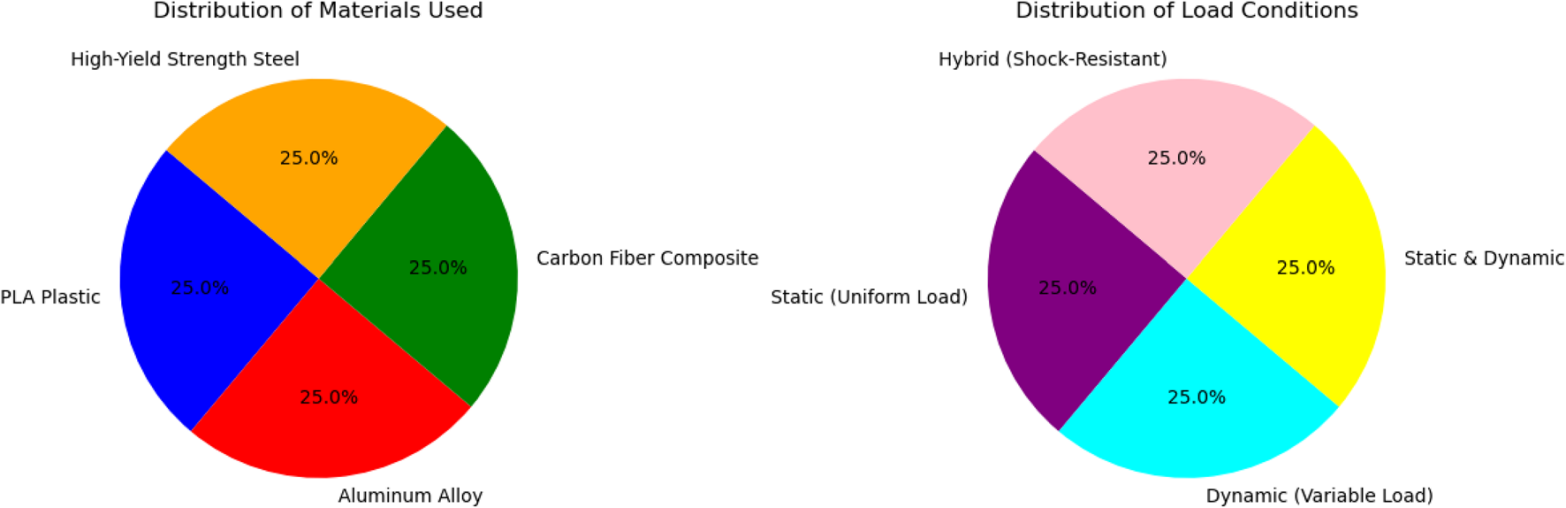
Material and load condition distribution in structural analysis.

**Fig. 33:**
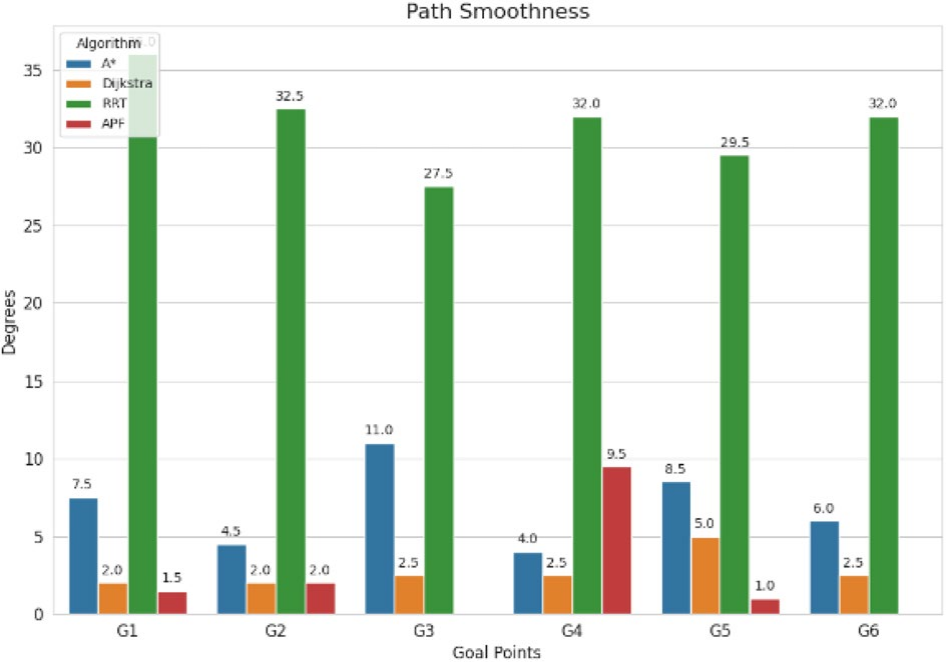
Path smoothness comparison.

**Fig. 34:**
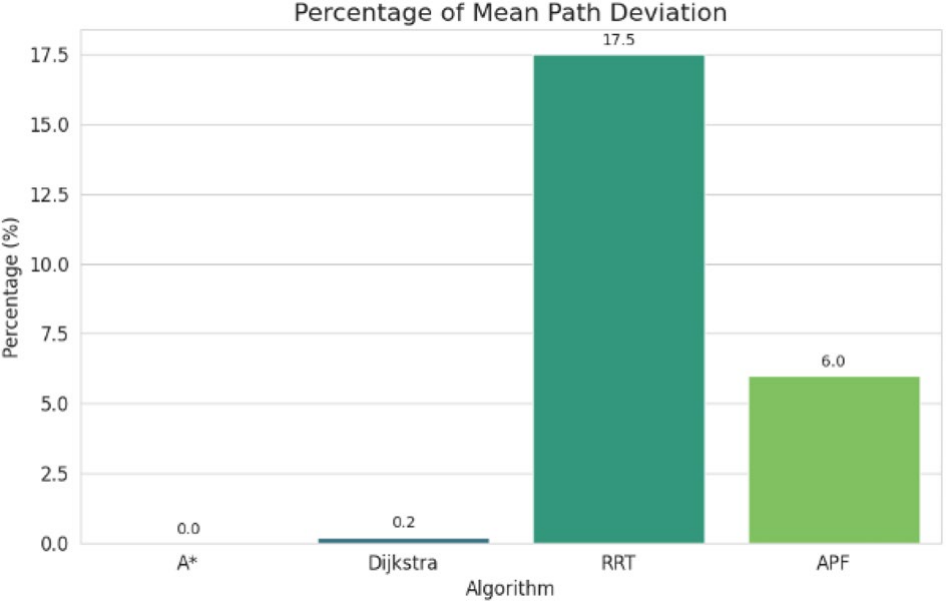
Mean percentage of path deviation comparison.

**Fig. 35:**
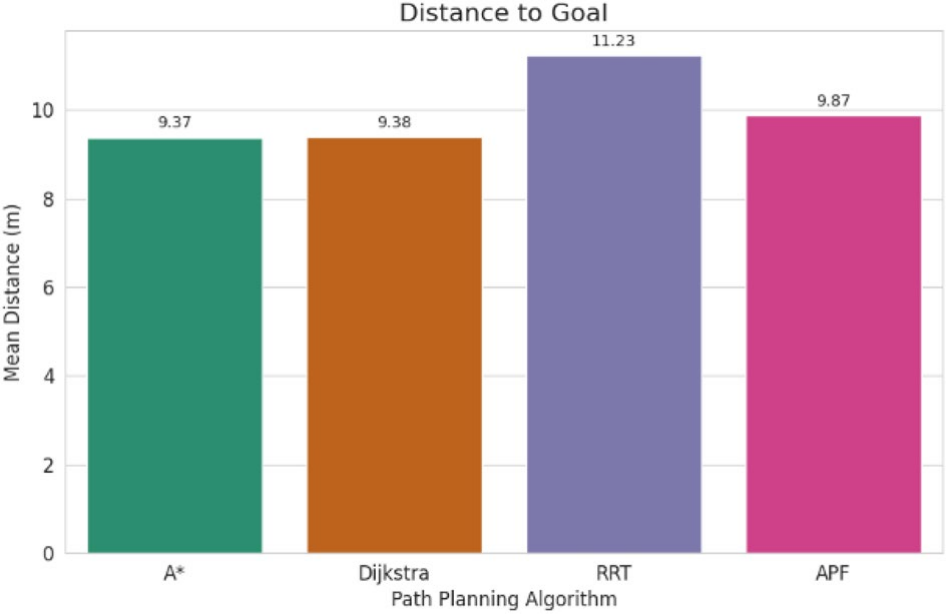
Path distance to goal comparison.

**Fig. 36:**
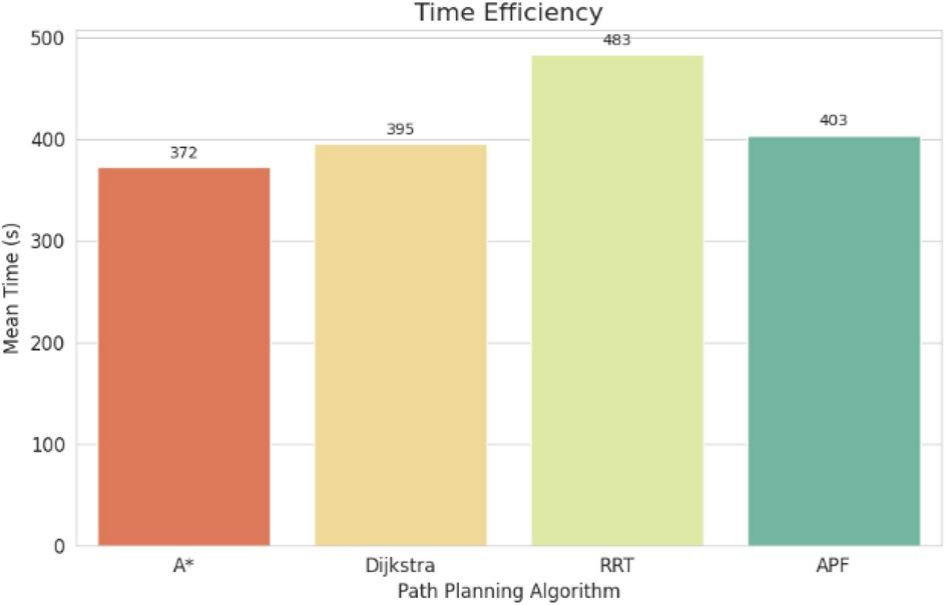
Time consumed comparison.

**Fig. 37:**
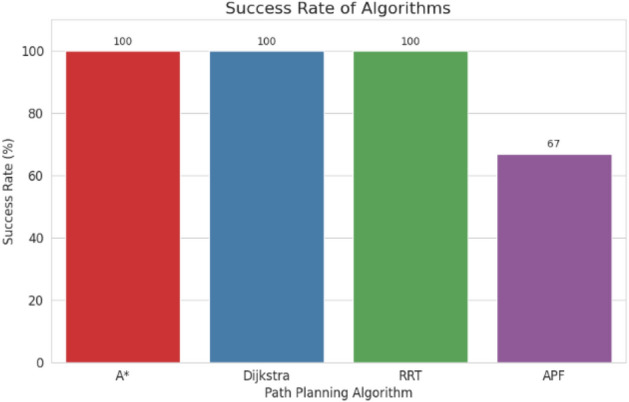
Success rate of algorithms.

**Fig. 38:**
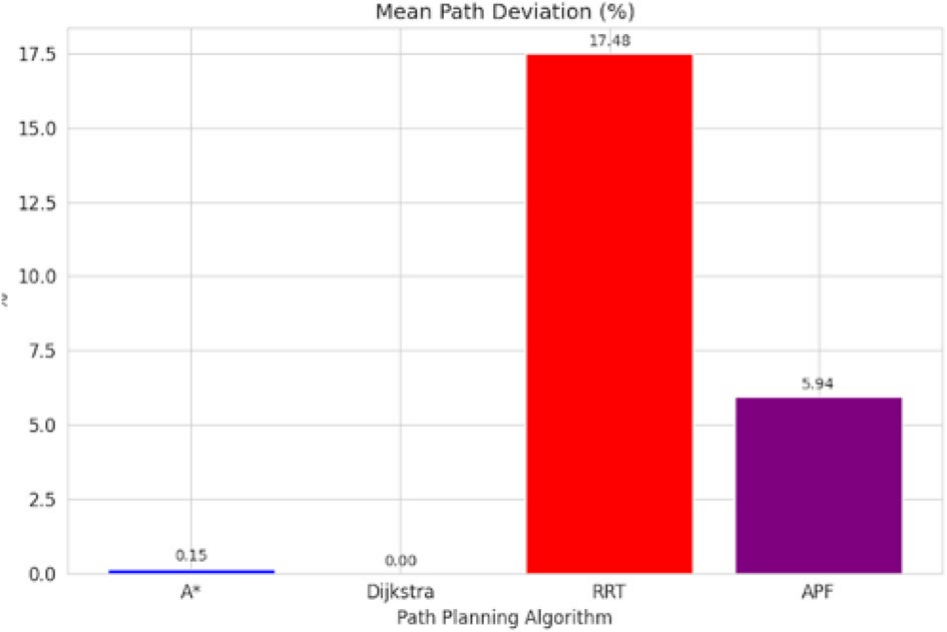
Mean Pat deviation of algorithms.

**Fig. 39:**
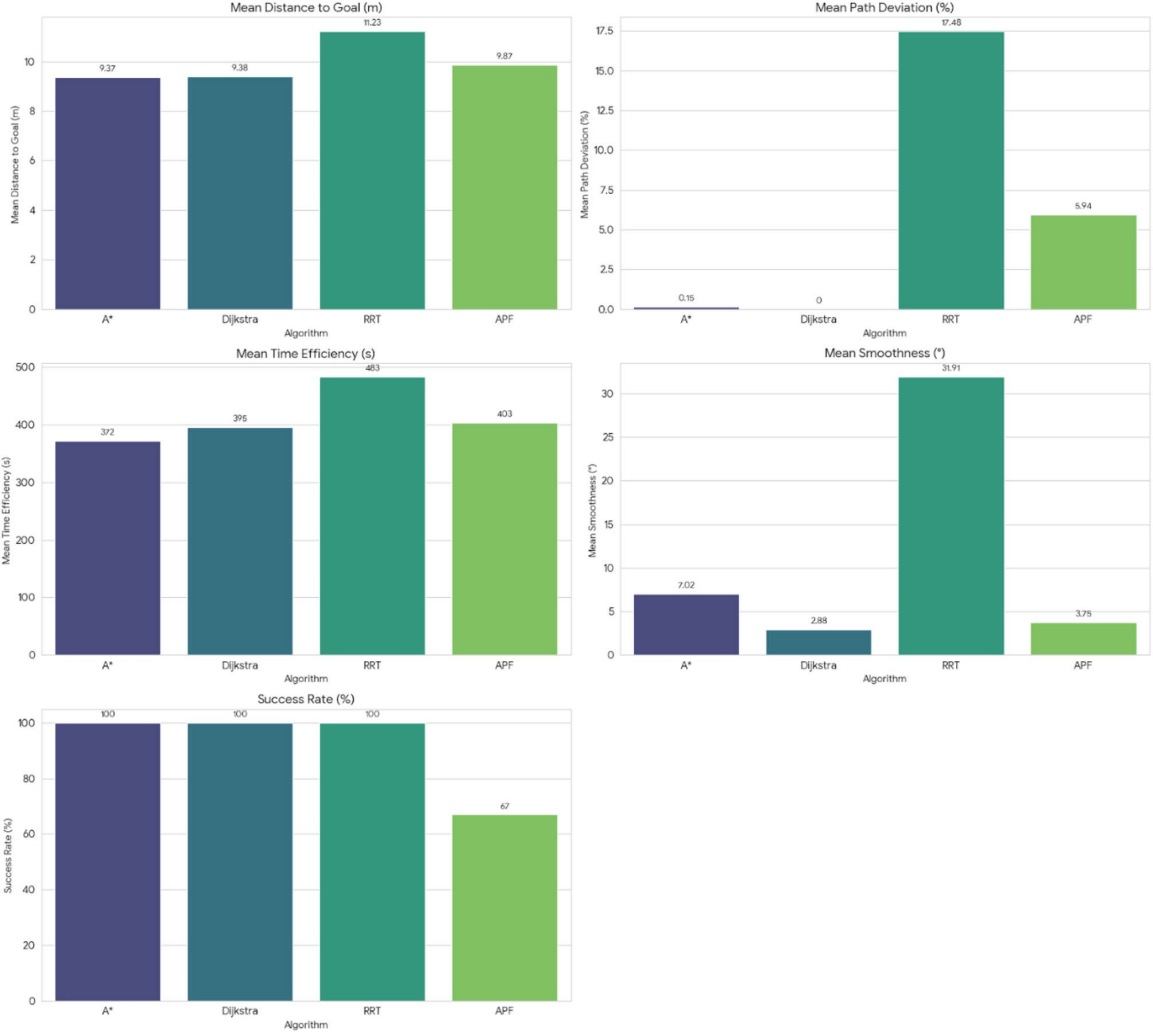
Comparison of path-planning algorithms.

**Fig. 40:**
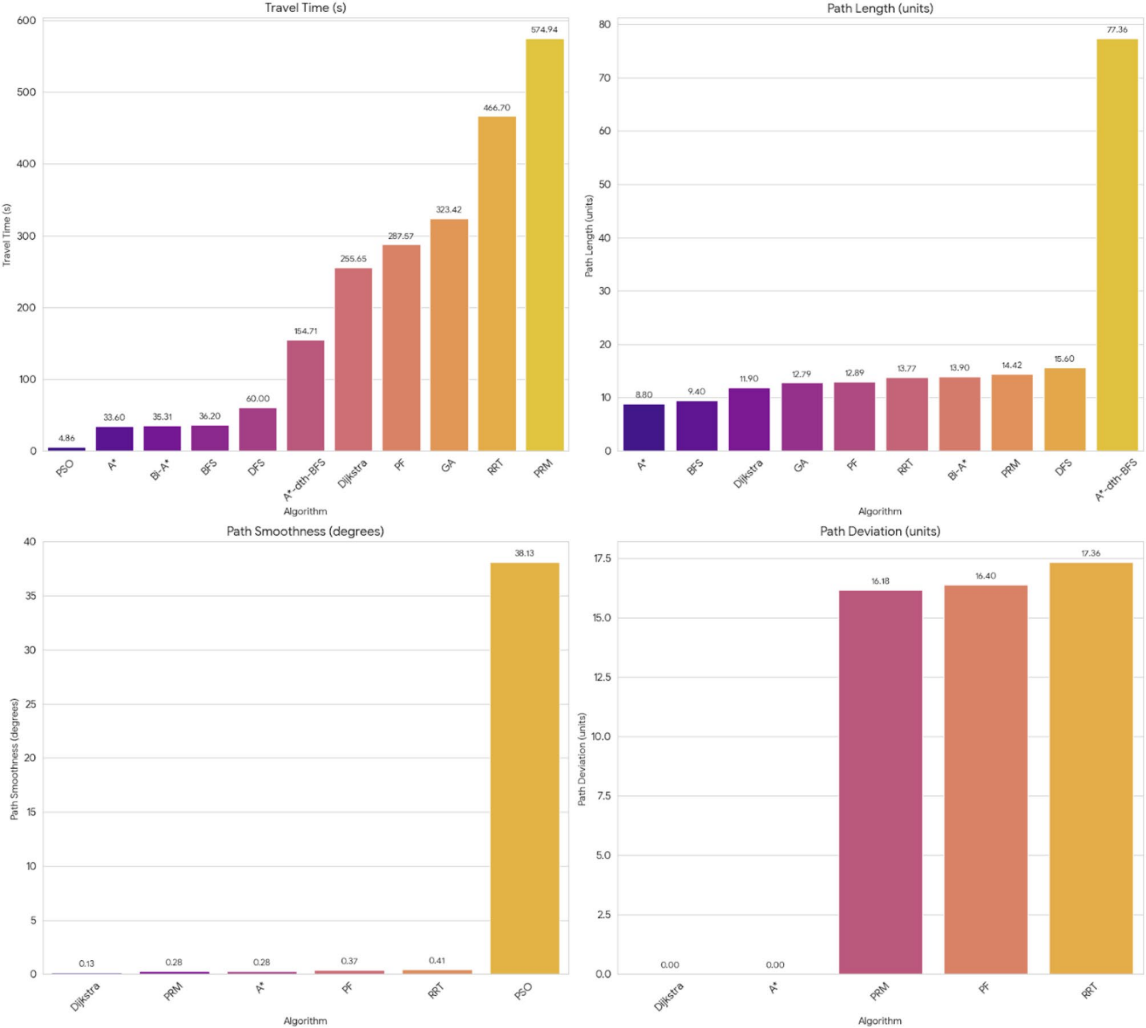
Comparative analysis of path planning algorithms based on travel time, path length, path deviation, and path smoothness.

**Fig. 41:**
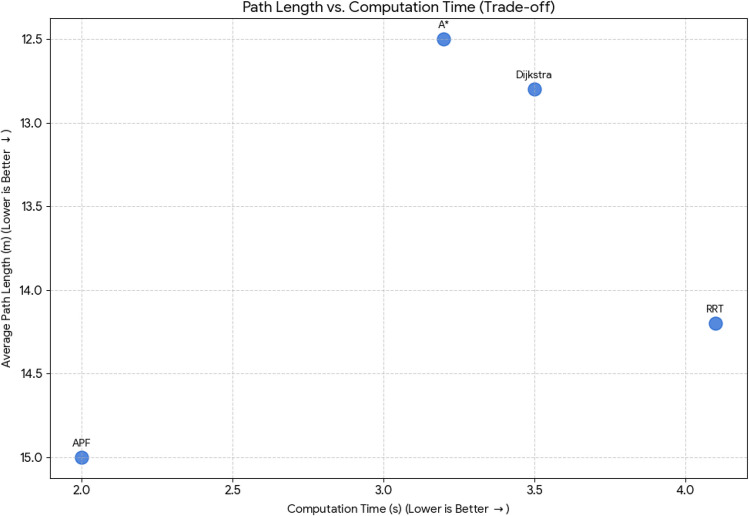
Comparative summary of path planners with respect to path length, computational cost, success rate, and smoothness*.*

**Fig. 42:**
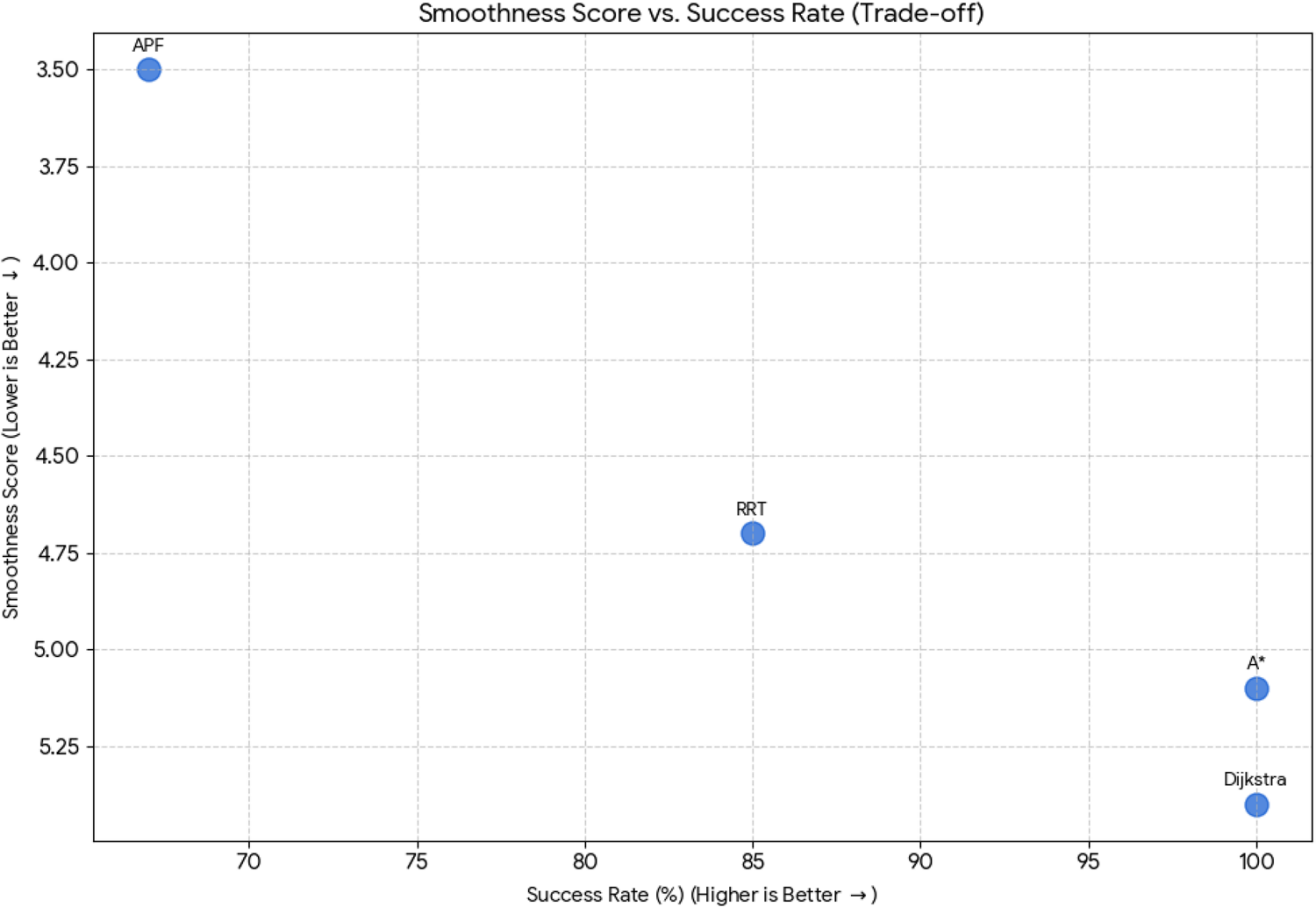
Comparative summary of path planning algorithms: smoothness vs. success rate comparison.

**Fig. 43:**
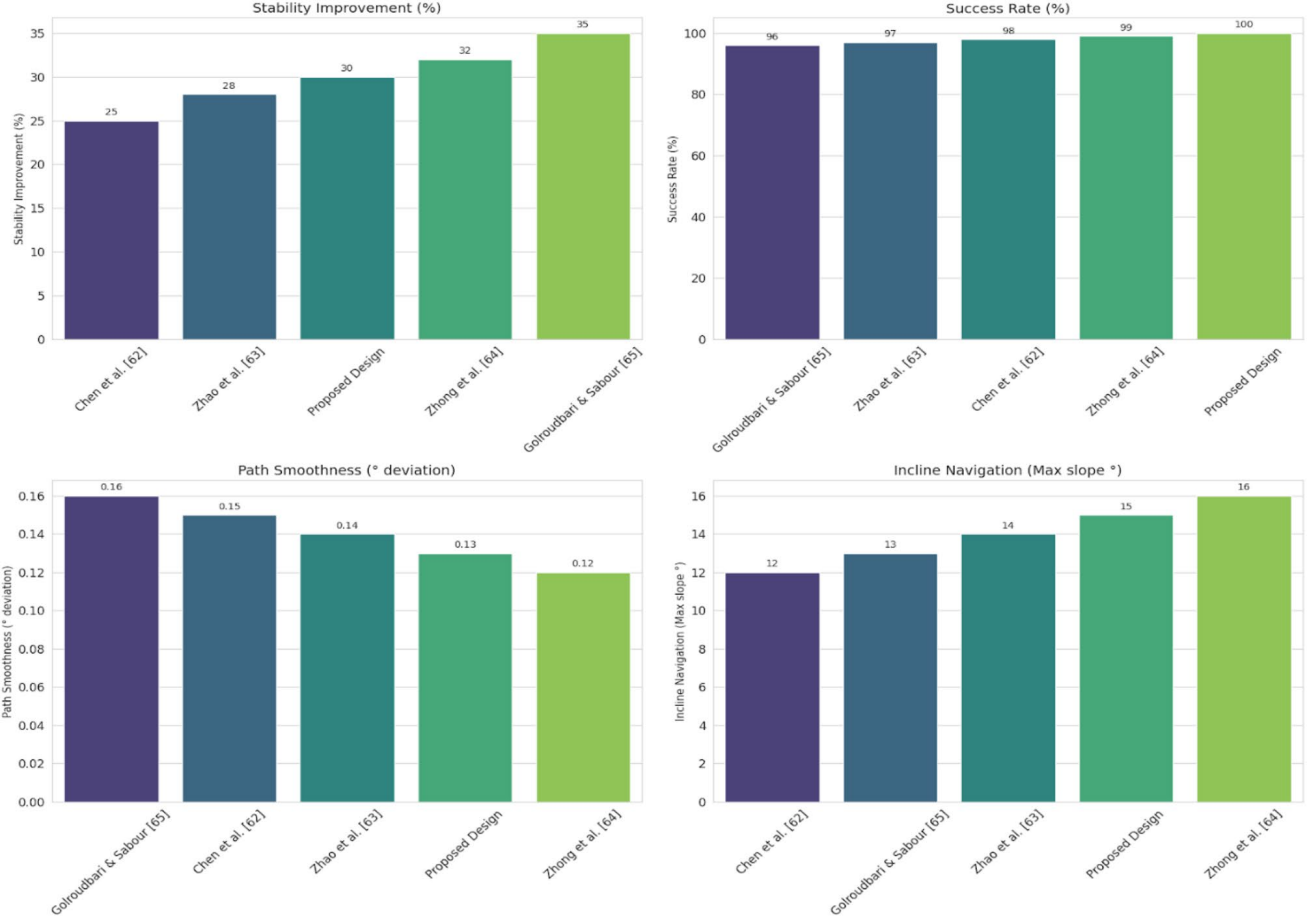
Quantitative comparison of hexapod locomotion performance metrics across recent designs and literature. Comparisons with earlier work show that combining stress analysis with locomotion algorithms improves long-term structural integrity.

**Fig. 44:**
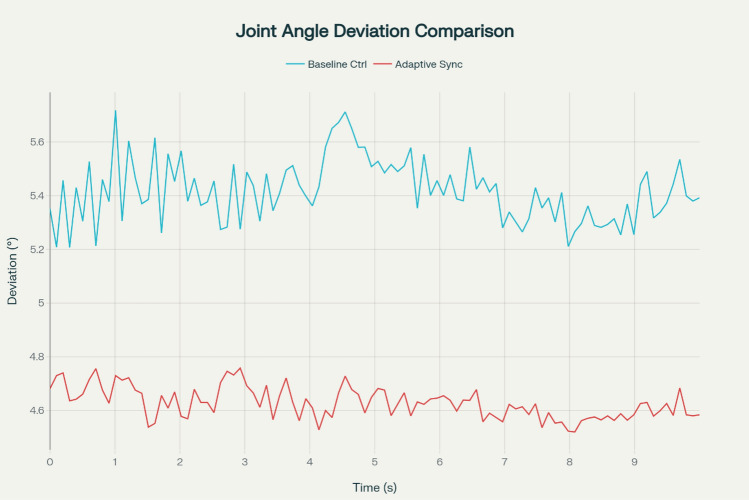
Improvement of the dynamic integration framework comparing to the standard.

Cohesive framework for enhanced mobility of the hexapod robot is evaluated comprehensively by analysing the stress distribution path planning efficiency and the joint leg synchronization. Results show that small PLA is a sensible solution for developing models. For improving the impact resistance later, the model is suggested with reinforced materials for the enhancement off the structure strength. Being A* and Dijkstra are better navigation approaches however a balance across the efficiency, exploration and safety, RRT and APF are considered as a good trade off techniques.

Further studies must focus upon combination reinforcement learning strategies that combine cognitive efficacy with sensor-based flexibility to ensure instant robustness in demanding contexts.

Hexapods efficiency is also evaluated based on various parameters which are given below such as energy consumption stability of the movement and the cost for the computation. All these parameters are very crucial to ensure the robot to consider in the real time implementation. The implementation of the adaptive dynamic control for the improved joint rigidity results in the diminishing value of oscillation by 12%. The previous one being conserves the energy and fixing the superfluous elements. Many path planning algorithms such as A* and Dijkstra will have high efficiency in normal operating conditions whereas they have a relatively more computational cost compared to RRT and APF techniques which are more cost effective, smooth and having reliable routes. The integration of the pad control technique locally and the adaptable synchronization proved to be a significant enhancement for the operational efficiency with substantial resource for computation. Future enhancements may involve hybrid learning-based planners to further optimize this trade-off dynamically.

## Conclusion

This work advances hexapod research by presenting an integrated framework combining structural finite element analysis, comprehensive multi-algorithm path-planning assessment, and biologically inspired adaptive joint synchronization, yielding enhanced multi-terrain navigation capability and structural optimization beyond prior isolated approaches. This work proposed an integrated scheme to optimize hexapod locomotion through structural design, path planning, and adaptive control. Structural analysis proved that a lightweight PLA construct could resist 10–20 N loads with efficient stress distribution and a safety factor of 2.8, although high-strength composites are still more resistant. Comparative performance of path-planning algorithms showed that A* and Dijkstra offer efficient and near-optimal navigation, A* and Dijkstra are as good as the optimal as with RRT, but at the expense of efficiency, and APF produces smooth paths but with limited success in cluttered space. Joint angle synchronization also improved stability, diminishing oscillatory deviations by about 12–15% on difficult terrain. The manuscript discusses that A* and Dijkstra’s algorithms produce near-optimal paths with 100% success but higher computational cost; RRT is computationally intensive with longer paths but reliable exploration; APF offers smooth paths with lower computational load but suffers from local minima leading to reduced success rate (~ 67%). The compromises made the study recommending a combination of techniques implementing are effective for real time navigation.

The research finds My balance between the computational cost and reliable navigation cool stop a hybrid modelling approach implementing both cognitive level strategies and statistical investigations suggested enhanced results. Furthermore, and intelligent articulation blended with good coordination for the joint angles suggested a reduction of more fluctuations and enhanced rigidity of the model on irregular terrains. This research gives a leading for that embodiment of biological inspiration for the development of models. Considering the finite element analysis to enhance the capacity of the robot, without making complex the motion of the model, a compact skeletal enhancement may be needed. Further research can be focus on the implementation of advanced machine learning techniques specific to sensor based adaptive control approaches facilitating the navigation of autonomous hexapods in complex environments can be considered. The current limitation of the model being the FEA analysis confining to the static loads in accessing the robot’s performance which is operated in different environments. Subsequent this research can go in the direction of interact to finite element analysis in simulating the effect of forces, impacts and other temporal variable loads, focusing on a greater comprehensive study of the hexapod strength.

The findings indicate that no single planner or design option is universally optimal across all scenarios. Robust hexapod guidance must achieve a compromise of rigidity, planner efficiency, and control flexibility. The next study will focus on hybridization of reinforcement learning controllers and sensor-driven adaptive adjustments to integrate the best features of conventional techniques with present-time training, facilitating implementation in unorganized and unpredictable contexts. The results add evident in demonstrating that none of the planner used for path search or any design methodology is universally optimal needs and every context. A robust hexapod navigation along with optimal design should have accomplished a greater trade-off between the flexibility the algorithm efficacy and the control technique. For their research will be carried on the combination of advanced reinforcement learning approaches blended with sensor driven dynamic adjustment to integrate the optimal parameters of the traditional methods with real time training and implementation in complex and unpredictable circumstances.

## Data Availability

All data that support the findings of this study are included within the article.
